# Plant Abiotic Stress Proteomics: The Major Factors Determining Alterations in Cellular Proteome

**DOI:** 10.3389/fpls.2018.00122

**Published:** 2018-02-08

**Authors:** Klára Kosová, Pavel Vítámvás, Milan O. Urban, Ilja T. Prášil, Jenny Renaut

**Affiliations:** ^1^Division of Crop Genetics and Breeding, Laboratory of Plant Stress Biology and Biotechnology, Crop Research Institute, Prague, Czechia; ^2^Department of Experimental Plant Biology, Faculty of Science, Charles University in Prague, Prague, Czechia; ^3^Environmental Research and Technology Platform, Environmental Research and Innovation, Luxembourg Institute of Science and Technology (LIST), Esch-sur-Alzette, Luxembourg

**Keywords:** stress dynamics, multiple stress treatments, stress-susceptible genotypes, stress-tolerant genotypes, protein isoforms and PTMs, functional studies

## Abstract

**HIGHLIGHTS:**

- Major environmental and genetic factors determining stress-related protein abundance are discussed.
- Major aspects of protein biological function including protein isoforms and PTMs, cellular localization and protein interactions are discussed.
- Functional diversity of protein isoforms and PTMs is discussed.

Abiotic stresses reveal profound impacts on plant proteomes including alterations in protein relative abundance, cellular localization, post-transcriptional and post-translational modifications (PTMs), protein interactions with other protein partners, and, finally, protein biological functions. The main aim of the present review is to discuss the major factors determining stress-related protein accumulation and their final biological functions. A dynamics of stress response including stress acclimation to altered ambient conditions and recovery after the stress treatment is discussed. The results of proteomic studies aimed at a comparison of stress response in plant genotypes differing in stress adaptability reveal constitutively enhanced levels of several stress-related proteins (protective proteins, chaperones, ROS scavenging- and detoxification-related enzymes) in the tolerant genotypes with respect to the susceptible ones. Tolerant genotypes can efficiently adjust energy metabolism to enhanced needs during stress acclimation. Stress tolerance vs. stress susceptibility are relative terms which can reflect different stress-coping strategies depending on the given stress treatment. The role of differential protein isoforms and PTMs with respect to their biological functions in different physiological constraints (cellular compartments and interacting partners) is discussed. The importance of protein functional studies following high-throughput proteome analyses is presented in a broader context of plant biology. In summary, the manuscript tries to provide an overview of the major factors which have to be considered when interpreting data from proteomic studies on stress-treated plants.

## Introduction

Stress can be defined as any environmental factor which adversely affects plant growth and development as well as crop quality and the final yield. Globally, the major abiotic stress factors—drought, high and low temperatures, and salinity—lead to the major reductions in crop yield. All plants, including crops, induce stress response leading either to stress escape, i.e., survival of stress treatment in metabolically inactive dormant stage such as seeds, or stress resistance, i.e., an active plant response to stress treatment. Stress resistance includes the strategies of stress avoidance, i.e., plant response aimed at maintenance of unstressed conditions at cellular and tissue levels, or stress tolerance, i.e., an active plant stress response to altered environment (Levitt, 1980).

The role of proteins in plant stress response is crucial since proteins are directly involved in shaping novel phenotype by adjustment of physiological traits to altered environment. The term “proteome” was introduced by Marc Wilkins in 1994 as protein complement of the genome, representing a whole of proteins in a given organism at a given time period. Unlike genome which is a static structure inherited from parents and defining plant genotype, changes in plant epigenome, transcriptome, proteome, and metabolome shape plant phenotype in response to both plant developmental and health stage as well as ambient environment. Proteins are directly involved in plant stress response both as structural proteins and also proteins involved in regulation of plant epigenome, transcriptome, and metabolome. Moreover, protein function is not dependent only on its molecular structure, but also on its cellular localization, post-translational modifications and interacting partners (Jorrín-Novo et al., 2009; Kosová et al., 2011).

**Figure 1:**
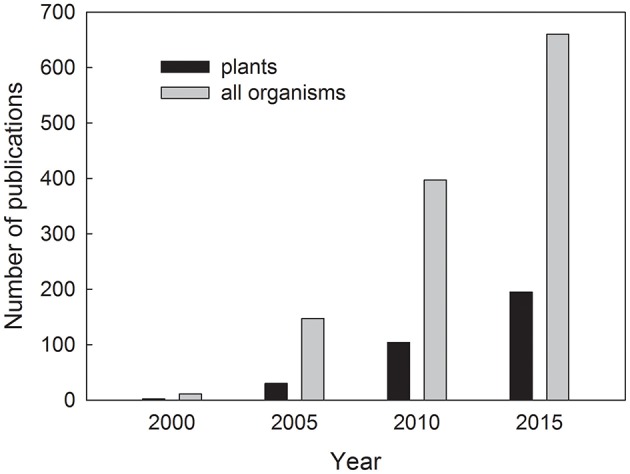
Number of publications found in Web of Science database as a reply to a query “plant proteome and stress” (black columns) for plants and “proteome and stress” (gray columns) for all organisms, respectively, for the years 2000, 2005, 2010, and 2015.

Plant stress proteomics is a dynamic discipline aimed at the study of plant proteome and protein biological functions in plants exposed to stress. The number of publications on plant proteome under stress has risen geometrically in the last 15 years (Figure 1). In recent years, more than 150 original research and review papers are being published on this topic each year, and it is practically impossible to provide an extensive summary of the whole issue in a single review paper. A total number of publications for “plant proteome and stress” amounts 1,634 according to Web of Science; October 23rd, 2017. This burst of proteomic studies is enabled by the advancements in high-throughput instrumentation techniques aimed at separation of complex protein mixtures and identification of the individual protein species as well as by advancements in genomics leading to publication of reference genome sequences for important crop species in recent years (summarized in Kosová et al., 2015). Moreover, several review papers on plant stress proteomics were published in the recent years including summarizing reviews on plant abiotic stress proteomics (Kosová et al., 2011), proteomics of major abiotic stresses such as drought, salinity and extreme temperatures (Ahmad et al., 2016), proteomics of low temperature stress (Janmohammadi et al., 2015; Johnová et al., 2016), dehydration stress (Johnová et al., 2016), heavy metal stress (Ahsan et al., 2009; Hossain and Komatsu, 2013), plant biotic stresses (Sergeant and Renaut, 2010) with a special focus on fungal pathogens (Rampitsch and Bykova, 2012a) and *Fusarium* head blight disease (Yang et al., 2013a). Moreover, specialized reviews were published on plant root proteome response to abiotic stress (Ghosh and Xu, 2014), plant post-translational modifications under abiotic stress (Wu et al., 2016), plant phosphoproteomics (Rampitsch and Bykova, 2012b), redox proteomics (Rinalducci et al., 2008; Mock and Dietz, 2016), S-nitrosoproteomics (Romero-Puertas et al., 2013), subcellular proteomics under stress (Hossain et al., 2012), chloroplast proteome under abiotic stress (Ning and Wang, 2016), crop proteomics (Salekdeh and Komatsu, 2007), plant proteome responses to salinity (Zhang et al., 2012; Kosová et al., 2013a,b), stress responses of major crops (Tan et al., 2017) including rice (Agrawal et al., 2009), maize (Pechanova et al., 2013), wheat and barley (Komatsu et al., 2014; Kosová et al., 2014), soybean (Wang and Komatsu, 2016; Yin and Komatsu, 2017), common bean (Zargar et al., 2017), Solanaceae species (Ghatak et al., 2017), stress proteomics of crops grown in temperate climate (Kosová et al., 2015), and others.

**Figure 2:**
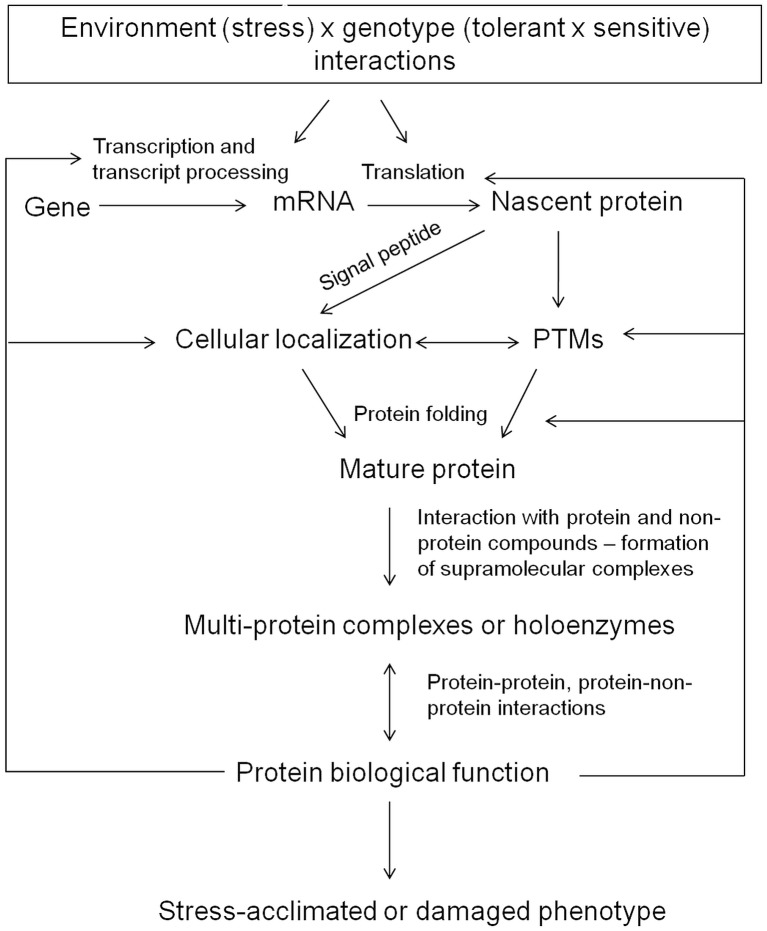
A schematic representation of the major factors determining protein accumulation and protein biological function.

Protein biological functions reflect diversity and versatility of life. The aim of this review is to summarize major factors determining stress-related protein accumulation as well as their cellular localization and final biological function. The crucial stress-related factors determining protein accumulation include the dynamics of the given stress treatment, the joint effect of multiple stress factors as well as genotypic background underlying differential gene expression between stress-tolerant and stress-susceptible genotypes. The major factors determining protein biological functions include protein cellular localization, protein posttranscriptional and post-translational modifications (PTMs), and protein interactions with other protein and non-protein compounds. Finally, functional studies should complement high-throughput proteome analysis and can thus contribute to uncover protein role in plant stress response. All these factors represent the major aims of plant stress proteomics studies and are discussed in the manuscript (Figure 2).

## Stress-related factors determining protein accumulation

### Environmental factors

#### Brief characteristics of the major abiotic stress factors

Generally, stress factors represent environmental constraints which induce disturbances in cellular homeostasis including imbalances in plant water regime (reduced water uptake by roots; dehydration stress) as well as imbalances in cellular metabolic pathways, especially aerobic respiration and photosynthesis, leading to enhanced formation of reactive oxygen species (ROS; oxidative stress). However, there are also several effects of the individual stress factors which are specific for the different stress factors (Kosová et al., 2011).

Drought means a decreased soil water potential which causes reduced water uptake by roots. Plant response at molecular level lies in osmotic adjustment, i.e., a decrease in osmotic potential of cell cytoplasm due to an enhanced accumulation of several osmolytes and hydrophilic proteins (e.g., LEA proteins). In leaves, drought leads to stomatal closure associated with reduced CO_2_ uptake resulting in imbalances between photosynthetic electron transport processes and carbon assimilation. As a consequence, cellular dehydration is also associated with enhanced ROS formation resulting in an induction of several ROS scavenging enzymes such as several thioredoxin (Trx) isoforms in drought-treated sugar beet (Hajheidari et al., 2007). At proteome level, drought-induced imbalances in cellular metabolism including photosynthesis result in alterations of several photosynthesis-related proteins (RubisCO large subunit, FBP aldolase) leading to an establishment of new homeostasis (Perlikowski et al., 2014).

At molecular level, heat as enhanced temperature causes enhanced kinetics of biomolecules leading to an enhanced risk of protein misfolding. Thus, plant response includes an induction of several heat-shock transcription factors (HSFs) and downstream heat-shock proteins (HSPs). Moreover, heat causes enhanced water evaporation from soil surface as well as enhanced leaf transpiration thus usually resulting in water deficit under field conditions. Heat thus also causes dehydration stress and oxidative stress (Kosová et al., 2015).

In contrast, low temperatures (cold and frost) cause decreased kinetics of biomolecules leading to reduced cell membrane fluidity and a decreased rate of enzymatic reactions. Frost leads to formation of ice crystals in soil thus leading to reduced water uptake by roots resulting in cellular dehydration. As a response, plant induces an accumulation of several osmolytes and hydrophilic proteins such as dehydrins. Temperature stress (both heat and cold) also leads to an imbalance between photosynthetic electrontransport processes and carbon assimilation processes resulting in enhanced photoinhibition and thermal energy dissipation (Kosová et al., 2015).

Salinity stress represents enhanced levels of salt ions in soil water solution. As a consequence of enhanced ion levels, decreased soil water potential reveals a so-called osmotic effect on plant cells which leads to an accumulation of several osmolytes in the process of osmotic adjustment. In addition, enhanced ions in plant water solution induce a so-called ionic effect which progresses with stress duration and leads to penetration of toxic ions such as Na^+^ into plant cells. Plant response lies either in active ion exclusion or intracellular compartmentation into vacuoles leading to tissue tolerance. Both processes require energy and are thus associated with enhanced levels of several ATP-dependent ion transporters such as Na^+^/H^+^-ATPases, V-ATPases, and inorganic pyrophosphatases (PPi ases). Osmotic effect is rapid and common to all dehydration stresses while ionic effect is time-progressive and specific to salinity stress (reviewed in Munns, 2002; Munns and Tester, 2008).

Flooding stress leads to anaerobiosis in plant roots thus inducing fermentation processes. Fermentation leads to an enhanced accumulation of organic acids resulting in acidic pH of cell cytoplasm which adversely affects activity of several cellular enzymes. Recently, Komatsu and her colleagues published several proteomic studies on soybean root tip response to flooding including studies with a focus on several cellular organelles (nucleus, nucleolus, mitochondria, endoplasmic reticulum, plasma membrane, cell wall). For example, alterations in U3 snoRNP ribonucleoprotein and NOP1/NOP56 complex indicating changes in ribosome biosynthesis and thus novel protein biosynthesis were found in nuclear proteome of soybean root tips at initial phases of flooding (Yin and Komatsu, 2015, 2016).

Heavy metal stress also reveals toxic ionic effects due to metal ion penetration into the cells resulting in either ion exclusion or vacuolar compartmentation as utilized in plant hyperaccumulators for phytoremediation (Ahsan et al., 2009; Hossain and Komatsu, 2013).

#### Dynamics of stress treatment, dynamics of stress response

Stress treatment as well as stress response are dynamic processes leading to an establishment of novel cellular homeostasis. According to Levitt (1980) and further researchers (see reviewed in Kosová et al., 2011), the following phases of plant stress response can be distinguished: an alarm phase, an acclimation phase, a resistance phase, an exhaustion phase, and a recovery phase or death. Each phase of plant stress response can be characterized by its unique proteome composition (Table 1). Therefore, it is important to precisely define environmental conditions and stress treatments applied in proteomic experiments. Moreover, regarding phenotype-based differential plant stress tolerance, it is becoming obvious that the same ambient stress conditions can cause differential stress response at cellular level as indicated by different plant-related physiological and biochemical characteristics. For example, different plants grown under the same conditions can significantly differ in physiological and biochemical characteristics such as tissue relative water content (RWC) or tissue Na^+^ levels under drought and salinity stresses, respectively (Flowers and Colmer, 2008; Tardieu, 2012). Stress adaptability therefore results from a complex combination of traits that influence ratio between supply and demand in a given environmental scenario (Passioura, 2012; Vadez et al., 2013). In agriculture, the major attention is paid to the effects of environmental stress on crop yield which impact, however, depends on the type of crop and growing purpose. An example of yield-related characteristics reflecting the effects of environmental stress can be drought-tolerance indices in wheat (Ali and El-Sadek, 2016). Proteomic studies have revealed that relatively low differences in ambient environments can result in profound alterations at proteome level if stress timing is well estimated. For example, a comparison of spring barley crowns grown under two levels of soil water capacity (30 and 35% SWC) led to differential response in proteins related to energy metabolism and protein degradation indicating an active plant acclimation upon 35% SWC while profound imbalances in energy metabolism and enhanced protein damage upon 30% SWC (Vítámvás et al., 2015). Therefore, in proteomic experiments, it is important (however, very often not mentioned at all) to provide relevant characterization of plant physiological and biochemical status not only by a description of ambient conditions, but also by providing plant-related characteristics (e.g., plant growth-related characteristics, phenological stage, plant and soil water regime-related characteristics, cellular levels of salt ions, mineral nutrients, heavy metals, etc.) reflecting stress impact at cellular level and different plant strategies to cope with stress (reviewed in Passioura, 2006; Tardieu and Tuberosa, 2010; Tardieu et al., 2011; Poorter et al., 2012a,b; Claeys et al., 2014; Blum, 2016). This creates a significant problem in detailed comparisons of published data or using proteomic meta-data analysis, especially in research comparing adaptable vs. susceptible genotypes.

**Table 1:** Brief characteristics of the individual phases of plant stress response (alarm phase, acclimation phase, resistance phase, exhaustion phase, recovery phase) with respect to plant stress tolerance, physiological response, and proteome response.

**Stress response phase**	**Alarm phase**	**Acclimation phase**	**Resistance phase**	**Exhaustion phase**	**Recovery phase**
Stress tolerance	Transient decrease	Increase	Maximum acquired stress tolerance with respect to the given conditions	Decrease	Decreased, but adjusted to altered environment
Plant physiology	Alterations in cell homeostasis - water regime, redox status	Plant response aimed at an adjustment to altered environment	Establishment of novel cell homeostasis in response to altered environment	Disruption of cell homeostasis (cell hydration, redox status)	Alterations in cell homeostasis in response to environmental changes
Protein response	Stress signaling and signal transduction from plasma membrane to nucleus Nucleus: Changes in gene expression Epigenetic modifications (DNA methylation, histone methylation and deacetylation)	Carbohydrate and Energy metabolism: enhanced catabolism and gain of immediately available energy ATP Alterations in protein, lipid, one-carbon, secondary metabolism - degradation of non-useful compounds from control environment and biosynthesis of novel compounds related to stress treatment Accumulation of stress-related compounds: chaperones, ROS scavenging enzymes, protective proteins (LEA, PRs)	Energy metabolism and cellular structures adapted to altered environment; most efficient levels of stress-protective compounds	Disruption of energy metabolism; Damage to cell structures;Proteasome-dependent protein degradation;Increase in PCD-related proteins	Signaling; Carbohydrate and Energy metabolism: enhanced anabolism and catabolism - photosynthesis, ATP metabolism Alterations in protein, lipid, one-carbon, secondary metabolism - degradation of non-useful compounds from control environment and biosynthesis of novel compounds related to altered environment Regulatory proteins: enhanced levels of proteins related to cell division and development progression

In the following text, brief characteristics of the individual phases of plant stress response at proteome level are given. An alarm phase can be characterized by initial stress factor treatment leading to disturbances in cellular homeostasis and thus inducing stress signaling resulting in alterations in gene expression. Changes in ambient conditions are sensed by receptors at plasma membrane inducing signaling pathways transferring stress signal from plasma membrane to nucleus and leading to the changes in gene expression. Unlike transcriptomic studies, only few proteomic studies focused on the investigation of the initial phases of plant stress response since relatively few changes were found at the proteome level at that phase. For example, *Arabidopsis* chloroplast proteome was investigated 1 day after the beginning of cold treatment (5°C), but only minimum alterations were found (Goulas et al., 2006). A decreased abundance of Ca^2+^ sensing receptor was found in maize chloroplasts after 4 h of salt treatment (Zörb et al., 2009). In contrast, alterations in several photosynthesis-related proteins (OEE1, PSB28, PSI type III chlorophyll *a*/*b* binding protein, Trx m-type) were found in cold-treated forage grass *Festuca pratensis* at 26 h of cold treatment (Kosmala et al., 2009). The susceptibility/adaptability of individual cultivars is connected to alarm phase, as very susceptible cultivars respond extremely on each environmental stimulus. The tolerant cultivars respond more slowly and can show later signs of stress (delayed stress onset; Lawlor, 2013). This different responsiveness strategy can likely be universal across all other phases.

An acclimation phase can be characterized by profound changes in protein metabolism including both protein biosynthesis and protein degradation resulting in enhancement of plant stress tolerance. During stress acclimation phase, alterations in gene expression lead to profound changes in several metabolic pathways (protein metabolism, carbohydrate metabolism, lipid metabolism, energy metabolism, secondary metabolism, phytohormone metabolism, etc.,) as well as an accumulation of stress-related proteins (proteins with chaperone and protective functions such as LEA, dehydrins; PR proteins, ROS scavenging and detoxification enzymes). Metabolic alterations are aimed at a synthesis of novel stress-related compounds while a degradation of several other ones. Alterations in energy metabolism are aimed at an enhancement of energy production in an immediately available form such as ATP since processes associated with an enhanced biosynthesis of stress-related compounds reveal enhanced energy requirements. An accumulation of several protective proteins, detoxification-related and ROS scavenging enzymes mitigates harmful effects of stress on cellular microenvironment such as increased dehydration and oxidative stress as well as increased amounts of toxic byproducts of cellular metabolism as a consequence of imbalances in cellular homeostasis (Kosová et al., 2011, 2015).

Resistance phase is characterized by achieving a maximum acquired stress tolerance under given ambient conditions. At molecular level, resistance phase can be characterized by efficient adjustment of cellular metabolism to altered ambient conditions as well as by sufficient levels of stress-protective proteins minimizing harmful effects of stress on cellular structures and metabolisms (Kosová et al., 2011).

Exhaustion phase is characterized by a decline in acquired stress response and can be achieved when stress treatment lasts too long or is too severe leading to exploiting plant resources necessary for maintenance of enhanced stress tolerance. Exhaustion phase can be characterized by rapid imbalances in cellular homeostasis, imbalances in cellular metabolism, transition from more efficient aerobic processes to less efficient anaerobic processes, and emerging signs of cellular damage including programmed cell death (PCD)-related changes (Kosová et al., 2011; Maršálová et al., 2016).

Recovery phase follows cessation of ambient stress treatment and can be characterized by an establishment of novel cellular homeostasis under non-stress conditions. Recovery phase is often overlooked in plant stress proteomics studies; however, it is equally important as stress treatment since efficient recovery affects further plant growth and development. During recovery phase, several stress-related compounds are degraded and novel compounds are synthesized, and these processes are associated with enhanced energy requirements. Enhanced abundances of 8 out of 12 glycolysis enzymes as well as photosynthesis-related proteins were found in drought-tolerant Australian spring wheat Excalibur at 24 h after rewatering indicating efficient restoration of energy metabolism after stress (Ford et al., 2011). In some cases of recovery, physiological parameters at whole plant level indicate return to control conditions while proteomic analysis reveals profound alterations in plant cellular environment under recovery with respect to control conditions prior to stress treatment as it was observed in chloroplast proteome of tomato plants subjected to 19 days of water withholding (drought stress) followed by 6 days of watering (recovery) (Tamburino et al., 2017). Decreased levels of proteins associated with photosynthesis including photosystem components (plastocyanin, chlorophyll *a-b* binding protein 4), Calvin cycle enzymes (PGK, PRK) and ATP synthase complex subunits (ATP β,γ) after 6 days of recovery indicate an ongoing decline in cellular energy metabolism following severe stress and a long way to reestablishment of cellular processes (Tamburino et al., 2017).

#### Stress treatments in the field: the impacts of multiple stress factors

Under field conditions, plants are usually exposed to combinations of multiple stress factors whose joint impact is not merely additive, i.e., it does not equal to the sum of the effects of the individual stress factors when acting separately (Mittler, 2006; Suzuki et al., 2014). Moreover, several stress factors also reveal secondary stress effects; for example, heat often leads to a dehydration stress since high temperatures enhance water evaporation and leaf transpiration. However, several different stress factors reveal some common features at cellular level. Under aerobic conditions, stress treatment leads to disturbances in cellular homeostasis resulting in enhanced ROS production (oxidative stress). Several abiotic stress factors also lead to reduced plant water uptake thus causing cell dehydration (dehydration stress). Drought can be accompanied by a strong oxidative stress due to photoinhibition of the components of photosynthetic electron transport chain. Flooding is associated with hypoxia (anoxia) leading to a shift in cellular metabolism from aerobic to anaerobic processes resulting in cytoplasm acidification due to an accumulation of organic acids as products of fermentation (Oh and Komatsu, 2015). Enhanced aluminum in soil is associated with soil acidity leading to changes in the soil mineral availability which results in an increased level of glutathione and other detoxification agents in rice roots (Yang et al., 2007).

At proteome level, the following stress factors comparisons either in different treatments or stress combinations within a single treatment were studied: a comparison of drought and salinity on salt-susceptible wheat cultivar Jinan 177 and a salt-tolerant *T. aestivum* × *Thinopyrum ponticum* hybrid Shanrong 3 (Peng et al., 2009), combined drought and heat treatments in Chinese arid shrub *Carissa spinarum* (Zhang et al., 2010), parallel and combined drought and heat treatments in a Syrian barley landrace Arta and an Australian cultivar Keel (Rollins et al., 2013), freezing combined with either drought or waterlogging in winter wheat (Li et al., 2014), a comparison of contrasting water stress treatments of drought and flooding on soybean seedlings (Oh and Komatsu, 2015).

Comparative proteome analysis of drought and salinity revealed larger changes in proteome composition induced by salinity than drought due to an ionic effect of salt stress (Peng et al., 2009). Similarly, heat caused larger alterations in proteome composition than drought in barley as well as in arid shrub *Carissa spinarum* due to the adverse effects of heat on plant photosynthetic apparatus (Zhang et al., 2010). In susceptible barley, heat led to a decrease in several photosynthesis-related proteins as well as an increase in thermostable RubisCO activase B isoform (Rollins et al., 2013). In relatively tolerant *Carissa spinarum*, heat induced several proteins involved in protection of photosynthetic apparatus such as thermostable RubisCO activase isoform, several sHSP and chaperonin proteins (Zhang et al., 2010). A comparison of the effects of two contrasting types of water stress—drought and waterlogging—on soybean root proteome revealed some similarities as well as differences in soybean root proteome response to these contrasting stresses (Oh and Komatsu, 2015). Both stresses induced an accumulation of glycolysis enzymes indicating an activation of carbohydrate catabolism and energy release; in addition, waterlogging induced fermentation enzymes and proteins related to cell wall modification while drought induced proteins involved in redox metabolism and alleviation of oxidative stress. Differential effects of these stresses can be found in S-adenosylmethionine synthetase (SAMS). In soybean roots, three isoforms of SAMS were found revealing differential dynamics in response to drought and waterlogging, respectively (Oh and Komatsu, 2015).

Moreover, a pretreatment with mild stress factor can help a plant to mitigate adverse effects of another stress. The corresponding effects deserve to be studied at proteome level. For example, mild drought or waterlogging applied prior to freezing can enhance freezing tolerance in winter wheat due to a positive effect of the mild stress on activation of cellular redox metabolism resulting in lower damage of photosynthetic apparatus under the subsequent freezing (Li et al., 2014). Similarly, a positive effect of an inoculation of barley plants with a mutualistic symbiont *Piriformospora indica* on mitigation of drought stress was found (Ghabooli et al., 2013). At proteome level, *P. indica* inoculation led to an increase in proteins involved in energy metabolism including photosynthesis as well as proteins involved in redox metabolism thus increasing barley tolerance to water stress (Ghabooli et al., 2013). Recently, a positive effect of arbuscular mycorrhizal fungus *Glomus masseae* on drought-treated wheat roots was reported leading to modulation of proteins related to sugar metabolism and cell wall rearrangement thus resulting in reduction of osmotic stress and maintenance of cellular integrity (Bernardo et al., 2017).

Summary: Abiotic stresses induce an active plant stress response at proteome level which is aimed at either maintenance of optimum cell environment (stress avoidance) or at an active acclimation to altered stress conditions (stress tolerance). Plant stress response is a dynamic process where several phases (alarm, acclimation, resistance, exhaustion, and recovery phase) with specific proteome composition can be distinguished. Relatively small differences in stress treatments can result in significant differences in plant proteome indicating either stress acclimation or stress damage. Combined stress treatments as occurring in the field reveal unique impacts on plant proteome which cannot be described as a mere sum of the distinct stress treatments.

### Genetic factors: stress-tolerant vs. stress-susceptible genotypes

Several proteomic studies focused on a comparison of proteome response to a given stress between a tolerant and a susceptible plant genotype or related species (Table 2, Supplementary Table S1A). Unfortunately, some authors often use genotypes with not well-documented differences at physiological level or they use only one physiological trait to describe it, making the result less usable in further studies. Simply, lower water use or higher biomass upon stress do not mean superior stress tolerance because of interactions between genotype × environment × management (Vadez, 2014; Vadez et al., 2014). For example, a comparison of drought-treated inbred vs. hybrid maize plants revealed an adverse effect of higher plant biomass and larger leaf area on a balance between photosynthesis and leaf transpiration rates leading to lower water use efficiency (WUE) and drought tolerance in the hybrid with respect to its parents (Holá et al., 2017). Comparison of changes in proteome composition under stress with respect to control conditions reveals higher numbers of differentially abundant proteins between control and stress conditions in stress-susceptible materials than in stress-tolerant ones. This indicates more profound disturbances in cellular homeostasis in the susceptible plants. For example, a comparison of differentially abundant proteins in salt-treated *Arabidopsis thaliana* vs. salt-treated *Thellungiella halophila* with respect to control conditions revealed 88 differentially abundant proteins in glycophyte *A. thaliana* while only 37 differentially abundant proteins in halophyte *T. halophila* under the same conditions (Pang et al., 2010). The possible reason for these differences between tolerant and susceptible species may lie in the fact that the given stress conditions do not cause such large disturbances in cellular homeostasis in tolerant halophytes than in susceptible glycophytes. The explanation of these differences may also lie in the fact that proteomic studies in stress-tolerant plants often identified constitutively enhanced abundance of several protective proteins, e.g., LEA proteins such as dehydrins, germin-like proteins GLP, universal stress protein USP (Vítámvás et al., 2010; Benešová et al., 2012; Kosová et al., 2013c) as well as several detoxification enzymes which efficiently remove toxic byproducts of cellular metabolism (Askari et al., 2006; Maršálová et al., 2016). Enhanced levels of ROS scavenging enzymes APX, Cu/Zn-SOD, and Mn-SOD were found in drought-tolerant maize and sunflower genotypes with respect to the susceptible ones, respectively (Benešová et al., 2012; Ghaffari et al., 2013), in salt-tolerant *T. aestivum* × *Lophopyrum elongatum* amphiploid with respect to its parental common wheat cv. Chinese Spring (Jacoby et al., 2013) as well as in differentially cold-tolerant winter wheats (Xu et al., 2013). In contrast, enhanced levels of APX and MDAR were found in salt-susceptible barley roots with respect to salt-tolerant ones since salinity treatment caused larger disturbances in redox homeostasis (both ionic and toxic effects of salt) in the susceptible genotype than in the tolerant one (Witzel et al., 2009). Differential levels of lactoylglutathione lyase (glyoxalase) involved in detoxification of methylglyoxal as a byproduct during glycolysis and threonine biosynthesis were found in barley root (Witzel et al., 2009) and barley crown (Maršálová et al., 2016) under salinity and in oilseed rape leaves under drought (Urban et al., 2017). Enhanced levels of cyanate hydratase involved in degradation of cyanate, a toxic byproduct of ethylene biosynthesis, were found in a halophytic plant *Suaeda aegyptiaca* (Askari et al., 2006). Due to constitutively increased levels of several stress-protective proteins in tolerant genotypes, a higher stress-inducible increase of some stress-responsive proteins such as HSP70 and thioredoxin h (Trx h) was found in susceptible genotypes with respect to tolerant ones under stress treatment (Manaa et al., 2011). Correspondingly, enhanced levels of stress-responsive transcription factors (TFs) such as NACα (Maršálová et al., 2016), bHLH (Vincent et al., 2007), and MYB-like (Wendelboe-Nelson and Morris, 2012) were found in tolerant plants with respect to the susceptible ones. Stress treatment thus causes larger disturbances in cellular homeostasis in susceptible plants than in tolerant (or stress-adapted) ones. Studies of physiological characteristics have shown that tolerant plants usually reveal also lower impacts of environmental stress factors on plant-related characteristics such as RWC (drought) or tissue Na^+^ content (salinity) with respect to the susceptible plants when exposed to the same ambient environment (Flowers and Colmer, 2008, 2015).

**Table 2:** Examples of major proteins (protein groups) revealing differential abundance between stress-susceptible and stress-tolerant plant genotypes. More details on proteomic experiments dealing with stress response in differentially stress-responsive genotypes are given in Supplementary Table S1A.

**Protein category**
Stress and defense (protective proteins): Chaperones: sHSPs (Majoul et al., 2004); HSC-70 (Pang et al., 2010); HSP90 (Manaa et al., 2011) COR/LEA: LEA-II dehydrins (Vítámvás et al., 2010; Benešová et al., 2012; Kosová et al., 2013c), LEA-III (Yang et al., 2013a); COR410 (Ford et al., 2011) Stress-responsive proteins: TSI-1 (Manaa et al., 2011); USP (Maršálová et al., 2016) Pathogenesis-related proteins: PR10 (Sugimoto and Takeda, 2009; Manaa et al., 2011; Guo et al., 2012); PR17 (Witzel et al., 2014) ROS scavenging: Mn-SOD (Xu et al., 2013); GS (Rasoulnia et al., 2011); APX, GPX, SOD (Janská et al., 2011); 2-Cys Prx (Vincent et al., 2007); Trx h (Manaa et al., 2011); LOX (Mostek et al., 2015) Detoxification enzymes: lactoylglutathione lyase (Witzel et al., 2009), cyanate hydratase (Askari et al., 2006); GST (Pang et al., 2010; Rasoulnia et al., 2011; Vítámvás et al., 2012)
Energy metabolism Glycolysis: FBP ALDO, TPI (Rasoulnia et al., 2011) Photosynthesis Antenna complexes: LHCB3 (Pandey et al., 2010); chl *a/b* binding apoprotein CP24 (Peng et al., 2009) Photosynthetic electron transport chain: OEC (OEE proteins OEE1, OEE2 (Taylor et al., 2005; Maršálová et al., 2016; Urban et al., 2017) ATP synthesis: ATP synthase CF1 β (Witzel et al., 2014) Carbon assimilation: RubisCO LSU and SSU, Calvin cycle enzymes PRK (Xu et al., 2013; Cheng et al., 2016) RubisCO associated proteins: RubisCO activase; carbonic anhydrase (CA) Chaperonins CPN60α,β (Guo et al., 2012; Urban et al., 2017) Mitochondrial respiration: ATP-synthase CF1β (Witzel et al., 2014); AOX (Camejo et al., 2013) Krebs cycle enzymes: mtMDH (Vítámvás et al., 2012; Xu et al., 2013); aconitase (Jacoby et al., 2013) γCA (Urban et al., 2017)
Gene expression Transcription factors: NACα (Maršálová et al., 2016); MYB-like (Wendelboe-Nelson and Morris, 2012); bHLH (Vincent et al., 2007)
Protein metabolism Protein biosynthesis: eIF3A - higher in T than S (Pang et al., 2010; Benešová et al., 2012) Ribosomal proteins: 60S ribosomal protein P2 (Maršálová et al., 2016) Protein degradation: 26S protease 6A (Maršálová et al., 2016)
S-adenosylmethionine (SAM) metabolism: SAMS (Faghani et al., 2015)
Hormone metabolism: DWARF3 (GA biosynthesis) (Wang et al., 2008) AOC, LOX2 (JA biosynthesis) (Pang et al., 2010)
Signaling: 14-3-3, G proteins (Alvarez et al., 2014; Faghani et al., 2015); small G-protein Rab2 (Wendelboe-Nelson and Morris, 2012) Regulatory proteins GLP3, GLP5a, GLP12 (Witzel et al., 2014) sGRP (Janská et al., 2011; Kosová et al., 2013d) PPR (Vincent et al., 2007)
Cell division/death and development
Cell division-related proteins: eIF5A isoforms (eIF5A1 vs. eIF5A2; Maršálová et al., 2016); ftsH protease (Pang et al., 2010) PCD-related proteins: TCTP (Mostek et al., 2015; Maršálová et al., 2016) Development-related proteins: lectin VER2 (Rinalducci et al., 2011a,b; Kosová et al., 2013d)
Structural proteins: Storage proteins: Legumin-like (Vítámvás et al., 2012) Membrane transport: annexin (Mostek et al., 2015); porin (Alvarez et al., 2014), V-ATPase (Mostek et al., 2015) Cell wall modification: β-expansin (Alvarez et al., 2014); CCOMT (Sugimoto and Takeda, 2009); COMT (Riccardi et al., 2004; Vincent et al., 2005)

*AOC, allene oxide cyclase; APX, ascorbate peroxidase; CCOMT, caffeoyl-coenzyme A O-methyltransferase; COMT, caffeic acid O-methyltransferase; FBP ALDO, fructose-1,6-bisphosphate aldolase; GLP, germin-like protein; GPX, glutathione peroxidase; GS, glutamine synthase; GST, glutathione-S-transferase; HSP, heat-shock protein; LOX, lipoxygenase; PPR, pseudoresponse regulator; PR, pathogenesis-related (protein); PRK, phosphoribulokinase; sGRP, small glycine-rich protein; SAMS, S-adenosylmethionine synthase; SOD, superoxide dismutase; TCTP, translationally-controlled tumor protein; TPI, triose phosphate isomerase; Trx, thioredoxin; TSI-1, tomato stress-induced protein 1; USP, universal stress protein*.

The differences in the impact of stress on tolerant and susceptible plants are also reflected in cellular metabolism. Larger disturbances in cellular homeostasis represent a higher risk of oxidative stress (ROS formation) thus adversely affecting aerobic metabolism, especially photosynthesis and ATP biosynthesis. A decrease in proteins of photosynthetic electron transport chain such as components of oxygen evolving complex (OEC) and photosystem II reaction center (RC PSII) as well as in RubisCO subunits and Calvin cycle enzymes was found in susceptible plants (Caruso et al., 2008; Gharechahi et al., 2014; Holá et al., 2017). In contrast, tolerant plants were able to adjust to altered environment and thus they revealed increased levels of crucial photosynthetic proteins such as OEC components of PSII as well as RubisCO subunits and RubisCO-related proteins such as RubisCO activase and carbonic anhydrase (Askari et al., 2006; Ge et al., 2012; Guo et al., 2012; Budak et al., 2013; Kausar et al., 2013; Witzel et al., 2014). Following RubisCO carboxylation activity, enhanced abundance of Calvin cycle enzymes was also found in tolerant plants. Similarly, an enhanced level of mitochondrial malate dehydrogenase (mtMDH) catalyzing dehydrogenation of malate to oxaloacetate was found in highly frost-tolerant winter wheat Mironovskaya 808 with respect to less tolerant winter wheat Bezostaya 1 under long-term cold (Vítámvás et al., 2012) as well as in salt-tolerant *T. aestivum* × *Lophopyrum elongatum* amphiploid with respect to its parental common wheat cv. Chinese Spring (Jacoby et al., 2013) indicating more efficient mitochondrial respiration in the more tolerant genotypes. Due to efficient elimination of oxidative stress and enhancement of aerobic metabolism, tolerant genotypes can cover enhanced demands on energy during active stress acclimation process and/or likely use this in more efficient recovery. These differences are reflected in enhanced levels of proteins involved in ATP biosynthesis, especially the components of mitochondrial or plastid ATP synthases, in tolerant plants (Cheng et al., 2016). In contrast, in susceptible genotypes, an enhanced risk of oxidative stress leads to disturbances in energy metabolism indicated by opposite patterns of different isoforms of proteins involved in energy metabolism such as glycolytic enzymes (Rasoulnia et al., 2011; Vítámvás et al., 2015). Moreover, an enhanced risk of oxidative damage can lead to a shift from aerobic metabolism to anaerobic fermentation processes which are, however, less efficient in ATP production.

Other genotypic differences in stress-treated plants include differences in proteins involved in several metabolic pathways such as protein metabolism including both protein biosynthesis and proteasomal degradation (Maršálová et al., 2016), proteins involved in S-adenosylmethionine (SAM) metabolism (Faghani et al., 2015) as well as proteins involved in hormone biosynthesis such as gibberellin (GA) (Wang et al., 2008) and jasmonic acid (JA) (Pang et al., 2010).

Moreover, differences between tolerant and susceptible plants subjected to stress treatments were also observed in regulatory proteins. Tolerant plants which were able to acclimate to stress conditions reveal enhanced levels of those regulatory proteins which contribute to maintenance of fully acclimated state or are involved in stimulation of cell division indicating restoration of plant growth and development. Stress treatments lead to alterations in the levels of several small glycine-rich proteins (sGRPs) which are involved in regulation of transcription and transcript processing including alternative splicing of mRNAs leading to synthesis of different protein isoforms under stress with respect to control. An increased abundance of several sGRPs was found in plants exposed to several abiotic stress factors such as in transgenic *Arabidopsis thaliana*, poplar, and *Nicotiana tabacum* under salt and flooding stresses, respectively (Kwak et al., 2005; Durand et al., 2010; Wang et al., 2012). Another example of regulatory proteins revealing genotypic differences represents a pentatricopeptide repeat protein (PPR) in two drought- and salinity-treated grapevines (Vincent et al., 2007); PPR proteins are known to localize to organellar genomes where they are involved in posttranscriptional regulation of gene expression due to their RNA binding activities (Barkan and Small, 2014).

A lower damage of plant tissues and lower energy costs on stress acclimation in tolerant genotypes in comparison to susceptible ones could also result in a relatively more positive effect on novel protein biosynthesis, plant growth, and development in tolerant genotypes compared to susceptible ones when subjected to stress. An increase in eIF3 and mitochondrial EF-TuM was found in drought-tolerant maize genotype CE704 subjected to 6 days of dehydration while a decrease in eEF1D was found in drought-susceptible maize genotype 2023 under the same conditions (Benešová et al., 2012). Differences in eIF5A3 level between salt-susceptible common wheat cultivar Jinan 177 and salt-tolerant *T. aestivum* × *Thinopyrum ponticum* introgression hybrid Shanrong 3 indicate a higher antisenescence ability of the hybrid with respect to its parental cultivar under salinity (Wang et al., 2008). In contrast, susceptible plants can reveal enhanced levels of apoptosis-related proteins indicating regulated degradation of damaged cells. Translationally controlled tumor protein (TCTP) is known to be involved in negative regulation of p53-induced apoptosis due to its binding to p53 leading to a downregulation of its activity (Chen et al., 2013). Genotypic differences in TCTP levels were found in differentially tolerant drought-treated barley cultivars (Mostek et al., 2015) as well as in salt-susceptible *H. vulgare* and salt-tolerant *H. marinum* under high salinity of 300 mM NaCl (Maršálová et al., 2016). Environmental stresses also profoundly affect plant development leading to regulation of timing of vegetative-to-reproductive phase transition. In temperate climates where long-term periods of low temperatures occur during winter several plants had evolved a requirement of a long-term period of low temperatures prior to their transition to flowering, a phenomenon known as vernalization (Sung and Amasino, 2005; Kosová et al., 2008). At proteome level, differential response to 21 day-cold treatment was found between a winter and a spring cultivar of common wheat. Results showed an accumulation of several regulatory proteins such as germin E and lectin VER2, involved in maintenance of vegetative phase in the winter wheat while an induction of proteins involved in transition to flowering in the spring wheat including eIF5A-2, glycine-rich RNA-binding protein, and adenine phosphoribosyltransferase involved in cytokinin biosynthesis (Rinalducci et al., 2011a,b; Kosová et al., 2013d).

Last but not least, genotypic differences were also found in structural proteins including storage proteins such as legumin-like protein in cold-treated winter wheat crowns (Vítámvás et al., 2012), membrane transport-related proteins such as annexin (Mostek et al., 2015), porin (Alvarez et al., 2014), V-ATPase (Mostek et al., 2015), and cell wall modification-related proteins such as β-expansin (Alvarez et al., 2014), and lignin biosynthetic enzymes caffeoyl-coenzyme A O-methyltransferase CCOMT (Sugimoto and Takeda, 2009) and caffeic acid O-methyltransferase COMT (Riccardi et al., 2004; Vincent et al., 2005).

However, it has to be kept in mind that “stress tolerance” vs. “stress susceptibility” are relative terms depending on the given environmental conditions and the dynamics of stress treatment. Moreover, an eco-geographic origin of the individual genotype or cultivar plays a significant role in the aspect of its phenotypic response. In fact, stress-tolerant and stress-susceptible genotypes can differ in biological strategies applied when to cope with stress and can even show opposite trends in mild vs. severe stress or for different developmental stages. Thus, any stress treatment should be based on previous detailed review of target population of environment (Tardieu, 2012) and application supported by hypothesis-driven occurrence (Vadez et al., 2013). For example, in winter oilseed rapes, the different water-related strategy (water-savers, water-spenders) influenced significantly the rate of water uptake from the pot, and, consequently, the differential protein abundances (Urban et al., 2017). In the water-savers group, proteins related to nitrogen assimilation, ATP metabolism and redox homeostasis increased under stress, while in the water-spenders category, carbohydrate/energy, photosynthesis, stress related and rRNA processing proteins were increased upon stress. However, both groups contain drought-adaptable cultivars. Thus, if it has to be decided which cultivar or genotype is “tolerant,” the rate of stress onset, duration of stress, and actual plant developmental stage have to be specified.

Examples of proteins revealing differential abundances between stress-tolerant and stress-susceptible plants is given in Table 2. More detailed information on the proteomic experiments carried out in genotypes with differential stress response is provided in Supplementary Table S1A.

Summary: Different plant genotypes or crop cultivars within a given plant species reveal differential adaptability to environment including stress treatments which is reflected by their differential ability to survive and recover following stress. Differential adaptability can be described by the terms of “susceptibility” vs. “tolerance,” respectively, which are, however, relative terms depending on the given genotypes and stress treatments. Proteomics approaches could help the researchers to uncover differences in plant stress response at molecular level prior than they can be detected at the whole plant level by determination of physiological characteristics. Recent studies revealed that at proteome level, tolerant genotypes reveal constitutively enhanced levels of several detoxification-related and stress-protective proteins thus it can be hypothesized that tolerant plants can better cope with stress-induced imbalances in cellular homeostasis than susceptible ones. As a consequence, stress treatment leads to lower level of oxidative stress in tolerant plants with respect to susceptible ones thus enabling the tolerant plants to efficiently adjust energy metabolism to enhanced demands of stress acclimation process. As a result, tolerant plants are able to efficiently acclimate to stress conditions and to restore their growth and development while susceptible plants reveal signs of cellular damage caused by adverse effects of stress.

## Stress-related factors determining protein biological function

The major factors determining protein biological function include cellular localization, posttranscriptional and post-translational modifications, and protein interactions.

### Protein cellular localization

**Figure 3:**
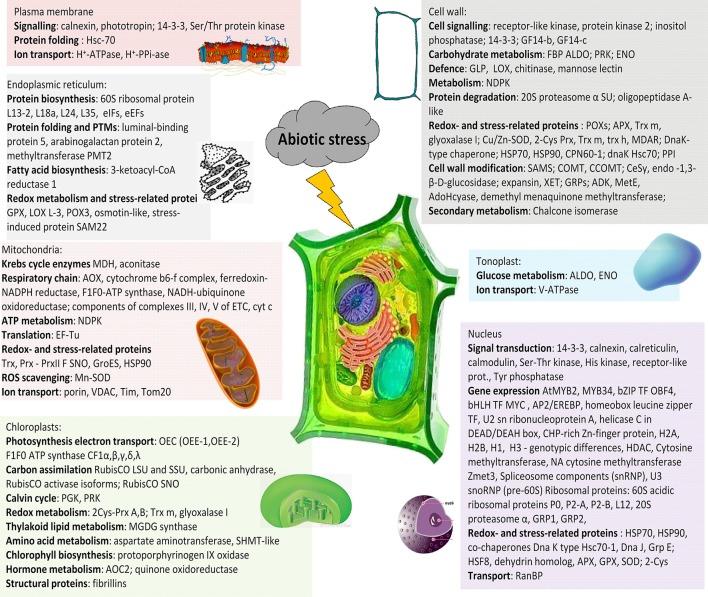
A schematic summary of the major stress-responsive proteins in the individual plant cell compartments. 2-Cys Prx, 2-cysteine peroxiredoxin; AOC, allene oxide cyclase; APX, ascorbate peroxidase; AOX, alternative oxidase; ENO, enolase; FBP ALDO, fructose-1,6-bisphosphate aldolase; GPX, glutathione peroxidase; GRP, glycine-rich protein; HSF, heat-shock (transcription) factor; HSP, heat-shock protein; LOX, lipoxygenase; MDAR, monodehydroascorbate reductase; MDH, malate dehydrogenase; OEC, oxygen-evolving center; PGK, phosphoglycerokinase; POX, peroxidase; PRK, phosphoribulokinase; SHMT, serine hydroxymethyltransferase; SNO, S-nitrosylation; SOD, superoxide dismutase; TF, transcription factor; Trx, thioredoxin.

Cellular fractionation represents an efficient means how to reduce complexity of cellular proteome. Separation techniques working with total proteome extracts usually do not ensure clear separation of all individual proteins present in the mixture leading to the situation that relatively low-abundant organellar proteins are overlaid by relatively high-abundant cytosolic proteins. Plant cell is composed of cell wall (extracellular matrix, ECM) as a part of apoplast and cytosol as a part of symplast connected with other cells via plasmodesmata. Apoplast and symplast are separated by plasma membrane representing a dynamic interface between these environments. Symplast contains organelles with double membrane envelopes such as nucleus, plastids and mitochondria, and vesicular compartments surrounded only by a single membrane such as components of secretory pathway including endoplasmic reticulum (ER), Golgi complex and trans-Golgi network (TGN), vacuoles, peroxisomes, glyoxisomes, and other vesicular structures. Each organelle plays a specific role in plant stress acclimation: nucleus is a site of stress signal transformation to gene expression, and chloroplasts and mitochondria are sites of aerobic metabolism, which are crucial for energy supply during stress acclimation. Classical approaches to cellular fractionation via differential centrifugation result in separation of four major cell fractions - nuclear, plastidial, mitochondrial, and microsomal. However, proteomic studies focused on organellar response to stress are still relatively scarce when compared to total proteome studies. A summary of the major proteins revealing alterations in their relative abundance under stress in the individual compartments is provided in Figure 3 (Table 3). More detailed information on proteomic experiments focused on subcellular proteomics in stress-treated plants is provided in Supplementary Table S1B and in specialized reviews on organellar proteomics under stress (Hossain et al., 2012).

**Table 3:** Examples of major proteins (protein groups) revealing differential abundance in cellular organelles (cell wall, plasma membrane, nucleus, chloroplast, mitochondria, tonoplast, endoplasmic reticulum) under stress.

**Proteins revealing differential abundance under stress identified in the given organellar proteome**
Cell wall (Extracellular matrix): Cell signaling: receptor-like kinase CHRK1, protein kinase 2; inositol phosphatase; 14-3-3; GF14-b, GF14-c (Pandey et al., 2010); Carbohydrate metabolism:,FBP ALDO; PRK, transaldolase, transketolase (Pandey et al., 2010), Defense: GLP, LOX (Komatsu et al., 2010); chitinase, mannose lectin (Bhushan et al., 2007); Metabolism: NDPK (Bhushan et al., 2007; Pandey et al., 2010) Protein folding: chaperones: DnaK-type chaperone; HSP70, HSP90, CPN60-1; dnaK Hsc70; PPI (Pandey et al., 2010); Protein degradation: 20S proteasome α subunit; oligopeptidase A-like (Pandey et al., 2010) Redox metabolism: peroxidases (linking of monolignols); APX, Trx m, glyoxalase I (Bhushan et al., 2007); Cu/Zn-SOD; glyoxalase I, 2-Cys Prx, Trx m, trx h, MDAR (Pandey et al., 2010); Cell wall modification: lignin biosynthesis (SAMS; COMT, CCOMT) (Zhu et al., 2007); cell wall polysaccharide metabolism (cellulose synthase, glucan endo-1,3-β-D-glucosidase (Bhushan et al., 2007); expansin, XET (Zhu et al., 2007); GRPs (Bhushan et al., 2007); ADK, AdoHcyase, SAM 2-demethylmenaquinone methyltransferase; 5-methyltetrapteroyltriglutamate-homocysteine methyltransferase (Pandey et al., 2010) Secondary metabolism: chalcone isomerase (Pandey et al., 2010)
Plasma membrane: Signaling: calnexin, phototropin (Hynek et al., 2009); 14-3-3, Ser/Thr protein kinase (Hüner et al., 2012) Chaperones: Hsc-70 (Hüner et al., 2012) Ion transport: H^+^-ATPase, H^+^-PPi-ase (Hynek et al., 2009; Komatsu et al., 2009)
Nucleus Signal transduction: 14-3-3, receptor-like protein kinase RLPK (Bae et al., 2003), calnexin, calreticulin, calmodulin (Subba et al., 2013); Ser-Thr kinase, His kinase, receptor-like protein, Tyr phosphatase (Bae et al., 2003) Gene expression: Transcription factors: AtMYB2, MYB34, bZIP TF OBF4, bHLH TF MYC (Subba et al., 2013); AP2/EREBP, homeobox leucine zipper TF (Bae et al., 2003) DNA- and RNA-associated proteins: U2 sn ribonucleoprotein A, helicase C in DEAD/DEAH box, CHP-rich Zn-finger protein (Subba et al., 2013); tRNA binding: glyceraldehyde-3-phosphate dehydrogenase (Pandey et al., 2008); DNA binding: aldolase (Pandey et al., 2008) Chromatin remodeling: Histone isoforms (H2A, H2B), alterations in H1, H3 - genotypic differences (Janská et al., 2011; Yin and Komatsu, 2015); HDAC (Bae et al., 2003) Cytosine methyltransferase (tolerant variety - (Janská et al., 2011); DNA cytosine methyltransferase Zmet3 (Komatsu et al., 2010) Transcript processing: Spliceosome components (snRNP) Ribosome biogenesis: U3 snoRNP (pre-60S) (Yin and Komatsu, 2015) Ribosomal proteins: 60S acidic ribosomal proteins P0, P2-A, P2-B, L12 (Subba et al., 2013) Protein degradation: 20S proteasome α (Subba et al., 2013) RNA processing: GRP1, GRP2 (Bae et al., 2003) Stress-related: chaperones (HSP70, HSP90), co-chaperones Dna K type Hsc70-1, Dna J, Grp E; HSF8 (Subba et al., 2013); dehydrin homolog Wcs66 (Bae et al., 2003) ROS scavenging: APX, GPX, SOD; 2-Cys Prx (Bae et al., 2003; Janská et al., 2011; tolerant variety); Transport: RanBP (Pandey et al., 2008)
Mitochondria: Krebs cycle enzymes (MDH, aconitase) (Camejo et al., 2013; Jacoby et al., 2013) Respiratory chain: AOX, cytochrome b6-f complex, ferredoxin-NADPH reductase, F1F0-ATP synthase, β (VHA-B);flooding: ↑Krebs cycle enzymes, NADH-ubiquinone oxidoreductase; ↓protein components of complexes III, IV, V of ETC, cyt c (Komatsu et al., 2012) ATP metabolism: NDPK (Camejo et al., 2013) Translation: EF-Tu (Komatsu et al., 2012) Chaperones: GroES, HSP90 (Jacoby et al., 2010) Redox homeostasis: Trx, Prx - PrxII F SNO (Komatsu et al., 2011) ROS scavenging: Mn-SOD (Camejo et al., 2013; Jacoby et al., 2013) Ion transport: porin, VDAC, Tim, Tom20 (Komatsu et al., 2012) Chloroplasts: Signaling: PAP (Tamburino et al., 2017) Photosynthesis electron transport: OEC (OEE-1, OEE-2) (Taylor et al., 2005) F1F0 ATP synthase CF1α,β,γ,δ,λ (Goulas et al., 2006) Carbon assimilation (RubisCO LSU and SSU, carbonic anhydrase, RubisCO activase (RCA) isoforms (Goulas et al., 2006); RubisCO SNO (Abat and Deswal, 2009) Calvin cycle: PGK, PRK (Goulas et al., 2006) Redox metabolism: 2Cys-Prx A,B; Trx m, glyoxalase I (Goulas et al., 2006) Thylakoid lipid metabolism: MGDG synthase (Zörb et al., 2009) Amino acid metabolism: aspartate aminotransferase, SHMT-like (Goulas et al., 2006) Chlorophyll biosynthesis: protoporphyrinogen IX oxidase (Zörb et al., 2009) Hormone metabolism: AOC2; quinone oxidoreductase (Goulas et al., 2006) Structural proteins: fibrillins (Taylor et al., 2005; Goulas et al., 2006; Urban et al., 2017)
Tonoplast: Glucose metabolism: ALDO, ENO (Barkla et al., 2009) Ion transport: V-ATPase (Barkla et al., 2009) Endoplasmic reticulum: Protein biosynthesis: ribosomal proteins (60S ribosomal protein L13-2, L18a, L24, L35; translation initiation and elongation factors - eIFs and eEFs Komatsu et al., 2012 Protein folding and PTMs: luminal-binding protein 5, arabinogalactan protein 2, methyltransferase PMT2 (Komatsu et al., 2012) Fatty acid biosynthesis: 3-ketoacyl-CoA reductase 1 (Komatsu et al., 2012) Redox metabolism and stress-related proteins: GPX, LOX L-3, POX3, osmotin-like, stress-induced protein SAM22 (Komatsu et al., 2012)

*More details on proteomic experiments focused on subcellular proteomics under stress treatments are provided in Supplementary Table S1B*.

*ADK, adenosine kinase; AdoHcyase, S-adenosyl-L-homocysteine hydrolase; ALDO, aldolase; AOC2, allene oxide cyclase 2; AOX, alternative oxidase; CCOMT, CCOMT-caffeoyl-coenzyme A O-methyltransferase; COMT, caffeic acid O-methyltransferase; CPN60, chaperonin 60 kDa; ENO, enolase; FBP ALDO, fructose bis-phosphate aldolase; GLP, germin-like; GPX, glutathione peroxidase; GRP, glycine-rich protein; HDAC, histone deacetylase; HSF, heat-shock factor; HSP, heat-shock protein; LOX, lipoxygenase; MDH, malate dehydrogenase; MGDG, monogalactosyl diacylglycerol; NDPK, nucleoside diphosphate kinase; PAP, polyphosphate-AMP phosphotransferase; POX, peroxidase; PPI, peptidyl-prolyl cis-trans isomerase; PRK, phosphoribulokinase; Prx, peroxiredoxin; SAM22, starvation-associated message 22 (protein); SAMS, S-adenosylmethionine synthase; SHMT, serine hydroxymethyltransferase; SOD, superoxide dismutase; Tim, mitochondrial inner membrane translocase; Trx, thioredoxin; Tom, mitochondrial outer membrane translocase; VDAC, voltage-dependent anion channel; XET, xylulose endo-transglycosylase*.

#### Cell wall

Cell wall, also known as extracellular matrix (ECM), represents not only apoplastic mechanical envelope of the cell, but also a dynamic structure actively involved in stress sensing and signaling. Plant stress proteomic studies dealing with ECM fraction were mainly focused on two contrasting stresses—dehydration (drought) and flooding. Extracellular matrix proteome in rice shoots under dehydration stress revealed alterations in proteins involved in stress signaling (nucleoside diphosphate kinase, NDPK, involved in γ-phosphate transfers and G-protein signaling), proteins involved in detoxification and ROS scavenging (APX, thioredoxin, glyoxalase I, chitinase), chaperones (DnaK, CPN60, and HSP20) as well as proteins involved in cell wall modification such as enzymes of phenylpropanoid biosynthetic pathway and methyltransferases involved in methylation of lignin components (Pandey et al., 2010). Moreover, several cytosol-related proteins were also identified in ECM including proteins involved in carbohydrate metabolism as pentose phosphate pathway (phosphoribulokinase, transketolase). They are proposed to be involved in biosynthesis of sugars as a part of osmotic adjustment under dehydration stress or to be involved in ROS scavenging due to production of NADPH (Pandey et al., 2010).

Alterations in proteome composition of soybean root cell walls exposed to flooding indicate a reduced cell wall lignification which is probably associated with decreased levels of Cu/Zn-superoxide dismutase (Cu/Zn-SOD), four germin-like proteins, lipoxygenases and glycoprotein precursors. The results indicate a suppression of lignification due to a downregulation of ROS and jasmonate biosynthesis under flooding (Komatsu et al., 2010). In contrast, enhanced levels of proteins involved in production of apoplastic ROS, namely H_2_O_2_, such as oxalate oxidase, germins, APX, Cu/Zn-SOD, Trx m were found in elongation zone of maize root cell walls (Zhu et al., 2007) as well as chickpea seedlings shoot cell walls (Bhushan et al., 2007) subjected to dehydration stress, respectively. Apoplastic ROS are involved in enhanced cell wall loosening necessary for elongation root growth as well as for cross-linking of monolignols and other phenolics during cell wall lignification process. Cell wall lignification is associated with an enhanced abundance of β-D-glucosidases involved in release of monolignols essential for lignin biosynthesis (Bhushan et al., 2007).

#### Plasma membrane

Plasma membrane is the primary site of stress sensing and its transformation into signaling. Alterations in ambient conditions are sensed by receptors at plasma membrane; for example, temperature decrease leading to alterations in membrane fluidity results in conformational changes in plasma membrane-located two-component histidine-type kinases (Murata and Los, 1997; Suzuki et al., 2000); or alterations in Na^+^ levels induce signaling associated with an active Na^+^ efflux via SOS1/SOS2/SOS3 complex (Zhu, 2002). Recently, several proteomic studies dealt with plasma membrane fraction under stress. Nouri and Komatsu (2010) investigated proteomic response of soybean plasma membrane to osmotic stress using both gel-based (2-DE) and gel-free (LC-MS/MS) approaches. Three H^+^-ATPase isoforms involved in ion efflux and revealing an increase under hyperosmotic stress were identified. The study has also identified several proteins regulating H^+^-ATPase activity including calnexin, one protein phosphatase, phototropin, and three isoforms of protein kinases. Similarly, several isoforms of H^+^-ATPase and H^+^-pyrophosphatase (H^+^-PPi-ase) were found in plasma membranes in aleurone layers of germinating barley embryos (Hynek et al., 2009). A study on plasma membrane fraction isolated from flooding-treated soybean indicated a role of 14-3-3 proteins and Ser/Thr protein kinase in regulating activity of plasma membrane H^+^-ATPase and maintenance of ion homeostasis (Komatsu et al., 2009).

#### Nucleus

Nucleus represents the major organelle involved in plant phenotype remodeling in response to environmental stress since it is involved in stress signal transformation into changes in gene expression. The study on nuclear proteome in drought-treated chickpea revealed the major protein functional groups belonging to gene expression, signal transduction, chaperones, chromatin remodeling, ROS scavenging enzymes, proteins involved in nucleocytoplasmic transport, and other regulatory proteins (Pandey et al., 2008).

Proteins involved in signal transduction include 14-3-3 proteins as well as secondary messengers involved in Ca^2+^ signaling such as calreticulin and calnexin. Alterations were found in several signaling- and regulatory-related proteins such as receptor-activated protein kinase C1 (RACK1), epsilon2-COP, beta-catenin, and others, revealing interactions with other proteins such as 14-3-3ζ, cAMP signaling pathway, and others determining the final protein function (Komatsu et al., 2014). Proteins involved in stress-induced gene expression include several ABA-dependent and ABA-independent transcription factors such as bZIP, bHLH, MYB, MYC, NAC, etc., (Bae et al., 2003). Proteins involved in chromatin remodeling include proteins involved in DNA methylation such as cytosine methyltransferase as well as proteins involved in histone PTMs such as histone deacetylase (HDAC) revealed alterations under stress (Pandey et al., 2008; Subba et al., 2013). It is also known that differential expression of various histone isoforms such as H2A (a canonical isoform H2A vs. a cold-responsive isoform H2A.Z) affects chromatin remodeling under low-temperature stress leading to transcriptome reprogramming (Janská et al., 2011). Differential H2A isoforms and decreased H1 and H3 were also found in nuclear proteome of soybean after the onset of flooding indicating profound chromatin remodeling (Yin and Komatsu, 2016). Nucleus also represents a site of formation of ribosomal subunits; therefore, several ribosomal proteins were identified among proteins responding to cold in *A. thaliana* nuclei (Bae et al., 2003). Several snoU3 RNA-associated proteins and NOP1/NOP56 complex which are involved in 60S ribosomal subunit biogenesis were found declined in flooded soybean indicating suppressed novel protein biosynthesis (Yin and Komatsu, 2016). Furthermore, stress also induced an accumulation of chaperones from HSP70 and HSP90 families to protect other proteins from disassembly under stress (Bae et al., 2003; Pandey et al., 2010). In addition, alterations in proteins involved in nucleocytoplasmic transport via nuclear pores such as Ran-binding protein (RanBP) were found under drought stress (Pandey et al., 2008).

Stress treatments also lead to alterations in protein PTMs. An analysis of nuclear phosphoproteins in soybean root tip during flooding led to identification of 27 phosphoproteins including zinc-finger/BTB domain-containing protein 47, glycine-rich protein, and rRNA processing protein Rrp5 which were regulated by ABA and phosphorylated in response to flooding (Yin and Komatsu, 2015).

#### Chloroplasts

Chloroplasts as sites of photosynthetic machinery are profoundly affected by stress treatments. Photosynthetic apparatus is very susceptible to alterations in cellular redox homeostasis affected by stress. Oxidative stress, especially a severe one such as ozone treatment, leads to decreased levels of components of photosynthetic apparatus including both photosynthetic electron transport chain and carbon assimilation proteins. In contrast, proteins involved in ROS scavenging and carbohydrate catabolism increased under oxidative stress which corresponds to decreased starch and increased sucrose levels (Ahsan et al., 2010; Hüner et al., 2012). The most crucial components of photosynthetic electron transport chain revealing alterations in response to stress are proteins of OEC complex involved in electron release from water, components of cytochrome b_6_f complex as well as components of electron acceptor complexes ferredoxin-NADP reductase and NAD(P)H-quinone oxidoreductase. Following electron acceptors, components of CF1-CF0 ATP synthase also revealed alterations under stress, especially the subunits of CF1 component including the subunits α, β, γ, ε, and λ. Stress also leads to alterations in proteins associated with carbon fixation including RubisCO subunits, RubisCO activase A and carbonic anhydrase. Alterations in photosynthetic electrontransport chain lead to profound alterations in chloroplast redox homeostasis and detoxification metabolism which result in enhanced levels of redox-related enzymes such as 2-cysteine peroxiredoxins (2-Cys Prx), thioredoxin m (Trx m) isoform, and lactoylglutathione lyase (glyoxalase I) levels as found in *Arabidopsis* chloroplast proteome under cold (Goulas et al., 2006). Alterations in crucial components of photosynthetic electrontransport chain such as the final electron acceptor ferredoxin-NADPH oxidoreductase and in subunits of CF_0_-CF1 ATP synthase complex were found in maize chloroplasts at early stages of salt treatment (Zörb et al., 2009).

Furthermore, stress also affects other plastidial functions such as plastidial glycolysis and proteosynthesis. Alterations in plastidial isoforms of GAPDH as well as plastidial translation elongation factor EF-Tu were found in wheat chloroplast proteome under salinity (Kamal et al., 2012). An increase in enzymes such as monogalactosyl diacylglycerol (MGDG) synthase involved in the biosynthesis of thylakoid membrane glycolipids were found in maize chloroplasts at early phases of salt stress indicating alterations in thylakoid membrane composition in response to stress (Zörb et al., 2009). Structural proteins are also affected by stress; as an example, fibrillins as the major protein components of plastoglobuli reveal alterations in response to drought in *Festuca arundinacea* (Kosmala et al., 2012), however, in oilseed rapes, fibrillins were found significantly accumulated in all cultivars upon drought (Urban et al., 2017). Last, but not least, protoporhyrinogen IX oxidase was found enhanced in maize chloroplasts under salt stress (Zörb et al., 2009); the enzyme catalyzes a conversion of chlorophyll precursor protoporhyrinogen which is known to act as a photosensitizer involved in ROS (singlet oxygen ^1^O_2_) production under stress. Enhanced protoporhyrinogen IX oxidase thus reduces the amount of protoporhyrinogen decreasing a risk of ROS formation (Zörb et al., 2009).

An analysis of tomato chloroplast proteome under drought stress and subsequent recovery revealed the importance of chloroplast as an environmental sensor of stress signals leading to specific chloroplast-to-nucleus (retrograde) signaling pathways and a crosstalk with nucleus-based (anterograde) ABA-dependent signaling network (Tamburino et al., 2017).

#### Mitochondria

Mitochondria as a major site of aerobic respiration face an enhanced risk of oxidative stress during stress acclimation. Therefore, enhanced levels of several ROS scavenging enzymes (Mn-SOD) were observed in mitochondrial proteome under stress (Taylor et al., 2005; Jacoby et al., 2010). Stress also led to enhanced levels of Krebs cycle enzymes such as malate dehydrogenase (MDH), components of F_1_F_o_ ATP synthase complex as well as alternative oxidase (AOX) which transfers electrons directly from ubiquinone pool to oxygen thus omitting cytochrome complexes of respiratory pathway (Jacoby et al., 2013). Unlike cytochromes, AOX is resistant to cyanide as a potential byproduct of plant metabolism under stress. Moreover, AOX activity prevents an overreduction of terminal electron acceptor thus preventing superoxide formation. Mitochondrial redox homeostasis is maintained by several thioredoxins (Trx) and peroxiredoxins (Prx). Besides ROS, NO can also modify several target proteins in mitochondria including ATP synthase CF1 β subunit, HSP90 and peroxiredoxins. It was found that S-nitrosylation of PrxII F in salt-treated pea resulted in a decrease of the protein biological activity (Camejo et al., 2013). Anoxia leads to increased levels of γ-aminobutyrate and tricarboxylic acid (TCA) cycle intermediates and, in contrast, to a decrease in components of mitochondrial electrontransport chain (ETC), especially complexes III, IV, and V, as found in soybean mitochondria under flooding (Komatsu et al., 2011). Stress also leads to altered levels of ion transporters such as voltage-dependent anion channel (VDAC), outer mitochondrial membrane porin (Jacoby et al., 2010), mitochondrial outer and inner membrane translocases Tom and Tim (Komatsu et al., 2011) as well as proteins with protective functions such as GroES chaperonin, chaperones and heat shock proteins in mitochondria thus preventing damage of protein complexes in respiratory chain (Taylor et al., 2005; Jacoby et al., 2013).

#### Endoplasmic reticulum

Endoplasmic reticulum (ER) is a major site of protein biosynthesis as well as folding and PTMs of nascent proteins. Investigation of ER-enriched fraction isolated from soybean root tips exposed to flooding revealed alterations in proteins involved in protein biosynthesis and PTMs, namely glycosylation, such as luminal-binding protein 5, arabinogalactan protein 2, and methyltransferase PMT2 (Komatsu et al., 2012). Moreover, an increase in 3-ketoacylCoA reductase 1 involved in fatty acid biosynthesis as well as in stress- and defense-related proteins such as stress-induced protein SAM22 (Starvation-associated message 22) and osmotin-like proteins were found. In addition, enhanced levels of proteins involved in anaerobic metabolism such as glycolysis (GAPDH, ENO) and fermentation (ADH1) as well as proteins involved in PCD induction (apoptosis-inducing factor homolog A) were found in ER-enriched fraction under flooding which is typical for anaerobiosis and aerenchyma formation (Komatsu et al., 2012).

#### Tonoplast

Vacuole plays an important role in plant salinity tolerance due to Na^+^ intracellular compartmentation. The rate of Na^+^ vacuolar sequestration is affected by the activity of tonoplast-located V-ATPase, which functions as an ATP-dependent Na^+^/H^+^ antiporter. In their study on *Arabidopsis los2* mutant lacking functional cytoplasmic enolase, Barkla et al. (2009) have shown a role of glycolytic enzymes aldolase (ALDO) and enolase (ENO) associated with tonoplast fraction for the activity of V-ATPase due to ATP supply.

Summary: Each cellular compartment reveals specific biological functions which correspond with specificities of their proteomes. Different protein isoforms located in different cellular compartements can reveal either analogous functions (e.g., cytosolic Cu/Zn-SOD, mitochondrial Mn-SOD, chloroplast Fe-SOD) or entirely different functions (e.g., cytosolic vs. nuclear enolase) depending on their interactions.

### Role of protein isoforms and post-translational modifications (PTMs)

Protein biological function is also determined by protein isoforms and post-translational modifications (PTMs). Protein isoforms differ in their sequence (primary structure) while PTMs represent chemical modifications of amino acid residues which can range from small molecules (NO) to whole peptides (ubiquitin, SUMO). Protein isoforms arise either from a single gene as products of posttranscriptional modifications such as alternative splicing, RNA editing, and others, or they can be products of orthologous or paralogous genes. Protein PTMs are reversible and can alter throughout protein life cycle, e.g., reversible phosphorylation of protein kinases or ubiquitination of proteins targeted to degradation. Protein isoforms and PTMs often differ in their molecular weight or isoelectric point making them distinguishable by 1-DE and 2-DE. Currently, over than 300 kinds of protein PTMs were described (Wu et al., 2016); however, only few of them were studied with respect to plant stress treatments. Stress-related PTMs mostly include phosphorylation, glycosylation, ubiquitination, sumoylation, and modifications induced by reactive molecular species (RMS) such as protein carbonylation and nitrosylation, etc., which result in significant alterations of protein function. Analysis of Web of Science publication database revealed that currently, the most studied protein PTMs upon stress represent phosphoproteomic studies (73 studies found as a reply for a query “plant phosphoproteome and stress” in October, 2017), followed by redox proteomics, glycoproteomics, and recently also by protein S-nitrosylation while other PTMs remain largely untouched in stress-treated plants.

Phosphoproteome analysis of drought-treated wheat seedling leaves revealed phosphorylated signaling proteins belonging to ABA-induced SnRK and PP2C signaling pathways, calcium-dependent protein kinase (CDPK), mitogen-activated protein kinase (MAPK) pathway, and calcium-dependent signaling proteins such as phosphatidylinositol-4,5-diphosphate. Other phosphoproteins identified include phosphorylated transcription factors such as ABA-dependent transcription factors ABI5, MYB1R1, and bHLH as well as ABA-independent transcription factors such as zinc finger CCCH domain containing proteins. Other phosphoproteins include proteins involved in transport such as aquaporins (AQPs) and proton ion pumps (H^+^-ATPases), proteins involved in stress defense such as zinc finger ABI5, three E3 ubiquitin ligases isoforms, ROS scavenging-related proteins glutamate decarboxylase 1 and glutathione peroxidase, and LEA proteins such as WCOR615, WCOR719 and WRAB17 (Zhang et al., 2014). Phosphorylation-regulated proteins involved in signaling and signal transduction also play important roles in plant-pathogen interactions affecting the extent of infection as revealed by a phosphoproteomic study on wheat infected with *Septoria tritici* (Yang et al., 2013b). Pathogen is sensed by plant receptor-like kinases and G proteins on plasma membrane. The initial signal is then transduced by MAPK and CDPK signaling cascades to nucleus where transcription factors such as WRKY involved in expression of genes associated with defense response are activated by phosphorylation. Similarly, alterations in phosphorylation patterns were also found in several signaling proteins produced by fungal pathogen such as protein kinase A, 14-3-3 protein, G protein subunit α, Ras GTPase, several MAPKs and an ABC transporter indicating that phosphorylation might play an important role in plant-pathogen interactions. Differential phosphorylation of dehydrin-5 protein and LEAIII proteins (WRAB17) was found in differentially-tolerant drought-treated common wheat and durum wheat cultivars, respectively (Brini et al., 2007; Zhang et al., 2014) which might indicate differential protein cellular localization between cytoplasm and nucleus (Rorat, 2006).

Redox proteomics: Possibilities of PTM modifications by reactive species of oxygen (ROS), nitrogen (RNS), carbonyl (RCS) and sulfur (RSS) are given in recent review by Mock and Dietz (2016). A proteomic study on three Iranian drought-treated wheat cultivars led to an identification of three different isoforms of thioredoxin h (Trx h) revealing different abundance patterns in response to drought stress as well as several other proteins known as potential targets of Trx h activity indicating an importance of plant redox response for protein protection under drought (Hajheidari et al., 2007). Apart from highly studied ROS, the effects of nitric oxide (NO) as a stress signaling molecule and the resulting S-nitrosylation of cysteine residues which affects protein biological activity becomes recently studied. For example, S-nitrosylation of RubisCO subunits in *Brassica juncea* exposed to cold leads to a decrease in RubisCO carboxylation activity (Abat and Deswal, 2009).

Protein glycosylation plays an important role in plant response to pathogens. Differential patterns of non-glycosylated vs. glycosylated isoforms of xylanase inhibitors with non-glycosylated forms increased more strongly than their glycosylated counterparts were found in wheat treated with *F. graminearum* Δ*Tri* mutant (Dornez et al., 2010). In wheat, DNA-damage inducible protein was found to undergo O-glycosylation under *F. graminearum* infection (Zhou et al., 2006). O-glycosylation may play an important role in response to low-temperature stress as reported for some dehydrin proteins isolated from cold-treated floral buds of blueberry (Levi et al., 1999) and pistachio (Yakubov et al., 2005); however, biological function of these glycosylated proteins remains unknown.

Examples of protein isoforms differential functions: Cellular redox metabolism, especially in chloroplasts and mitochondria, represents a finely tuned network of redox reactions, which are aimed at minimizing harmful damage by RMS, especially under stress. Cellular redox homeostasis thus represents a result of a coordinated action of several isoforms of redox proteins with specific, but overlapping functions enabling fine tuning of redox metabolism. For example, more than 20 Trx and Trx-like protein isoforms (Trx-f1, Trx-f2, Trx-m1-4, Trx-x, Trx-y1, Trx-y2, Trx-z, atypical Cys-His rich Trx), NTRC (NADPH-dependent Trx reductase C), protein disulfide isomerase-like (APR-1-3), thiol-based peroxidases and ascorbate peroxidases were identified in *A. thaliana* chloroplasts (Meyer et al., 2005; Dietz, 2016). The individual redox proteins are involved in reactions with thiol groups of target proteins in photosynthetic electron transport chain thus revealing specific, but overlapping functions which are still far from being fully understood (König et al., 2012). Similarly, several small HSP proteins (sHSPs) with overlapping chaperone functions were identified in heat-treated wheat grains (Skylas et al., 2002; Majoul et al., 2004) indicating an enhanced need of protein protection against heat-induced damage.

Other protein isoforms may differ in their tissue localization or induction by different stresses. Three isoforms of SAMS were identified in soybean revealing differential patterns in relative abundance both at transcript and protein levels in different seedling parts (root, cotyledon, hypocotyl) under drought and flooding, respectively (Oh and Komatsu, 2015; Wang et al., 2016). SAMS is known to be involved in methylation of lignin components; thus, the different abundances of different SAMS isoforms may underlie differences in plant cell growth and lignification of cell walls.

It is known that protein enzymatic activity depends on temperature. Therefore, alterations in protein isoforms of several enzymes differing in their thermostability were found on 2-DE (2D-DIGE) gels under temperature stress including both high and low temperatures. In *Arabidopsis* chloroplast fractions under cold, alterations in relative abundance of RubisCO large and small subunits were found under cold with respect to control indicating alterations in isoform composition of the whole RubisCO holoenzyme thus affecting its final activity under altered temperature (Goulas et al., 2006). Alterations in protein isoform composition become obvious, especially under heat. For example, cereals from the tribe Triticeae encode two RubisCO activase isoforms - a conventional RubisCO activase A and a thermostable RubisCO activase B. In heat-treated barley, thermostable RubisCO activase B revealed an increase while thermo-unstable RubisCO activase A revealed a decrease upon the same treatment (Rollins et al., 2013). A study in *Chenopodium album* revealed a higher thermostability of chloroplast APX and Cu/Zn-SOD isoforms than mitochondrial APX and Mn-SOD isoforms (Khanna-Chopra et al., 2011).

Protein isoforms can reveal either similar or entirely different biological functions. A few examples of distinct biological functions of protein isoforms are given below. Eukaryotic translation initiation factor eIF5A in cytosol is known to act as a translation initiation factor involved in regulation of protein biosynthesis. However, its nuclear isoforms eIF5A1 and eIF5A2, differing in hypusination level, were reported to differentially regulate cell cycle leading to either apoptosis (PCD) or cell division (Thompson et al., 2004). In a comparative proteomic study on salt-treated glycophytic *H. vulgare* and halophytic *H. marinum*, different eIF5A isoforms were found with eIF5A1 isoform in *H. vulgare* while eIF5A2 isoform in *H. marinum* (Maršálová et al., 2016). The presence of eIF5A1 in *H. vulgare* corresponds with enhanced levels of several apoptosis-related proteins found in *H. vulgare* under high salinity. Flooding stress-related translocation of cytochrome c from mitochondria into cytoplasm via VDAC leads to cytochrome c interaction with caspases and induction of PCD (Komatsu et al., 2011).

Similarly, nuclear isoforms of cytosolic proteins FBP aldolase, enolase, and glyceraldehyde-3-phosphate dehydrogenase (GAPDH) known as glycolytic enzymes are involved in important regulatory processes in nucleus. Nuclear isoform of fructose-bisphosphate aldolase (FBP aldolase) is known to act as a DNA-binding protein involved in regulation of expression of its own gene as well as other genes (Ronai et al., 1992). Nuclear isoform of GAPDH is known to act as a tRNA-binding protein involved in tRNA export (Singh and Green, 1993). It has also been reported that FBP aldolase may be involved in an integration of intra- and extracellular signals associated with growth, development, and sugar biosynthesis (Li et al., 2012) while non-phosphorylating isoform of GAPDH may be involved in regulation of ROS levels (Bustos et al., 2008). Nuclear isoform of enolase encoded by *Los2* locus in *A. thaliana* functions as a transcriptional repressor of STZ/ZAT10 which is a repressor of cold-inducible CBF pathway; nuclear isoform of enolase thus acts as an indirect positive regulator of CBF pathway and CBF-regulated *COR* gene expression (Lee et al., 2002). Tonoplast-associated isoforms of aldolase and enolase are involved in activation of V-ATPase in salt-treated *Mesembryanthemum crystallinum* plants leading to Na^+^ vacuolar accumulation (Barkla et al., 2009). It can thus be concluded that biological function of a given protein is determined by PTM as well as cellular localization.

### Functional proteomics and interactomics: from proteome description to protein biological function

Proteomic analysis enables the researchers to identify proteins, which reveal quantitative or qualitative differences between control and stress treatments. These proteins might play a crucial role in plant stress response. Biological function of a given protein is dependent on its PTM, cellular localization as well as interaction partners. Therefore, functional studies of these proteins are required to characterize their role in plant stress response. Functional studies using knock-out mutants, enzyme assays, tools for protein-protein interaction study, etc., provide valuable data on protein biological functions which can complement the data on protein relative abundance obtained by high-throughput proteomic analysis.

In winter cereals, transition to flowering is regulated by a period of low temperatures, a phenomenon known as vernalization. An interactomics study in winter wheat using two independent methodical approaches, yeast two-hybrid GAL4 system and split-GFP fluorescent complementation in *Nicotiana benthamiana*, has confirmed physical interactions between proteins involved in flowering regulation (TaVRT1/VRN1, TaVRT2, VRN2, TaFT1/VRN3, TaHd) as well as between other protein groups such as proteins involved in signaling (two phospholipases C, a receptor-like protein kinase, G protein) and microtubule remodeling (α-tubulin and TaTil, a lipocalin known to be involved in microtubule polymerization) which opens new ways to study their functions under cold (Tardif et al., 2007).

Protein interactions play also crucial roles in plant-pathogen interactions. For example, wheat treatment with *Fusarium graminearum* Δ*Tri* mutants led to changes in wheat xylanase inhibitors (Dornez et al., 2010). *Fusarium graminearum* Δ*Tri* mutants are unable to produce mycotoxin deoxynivalenol (DON) thus they are less infectious than wild type pathogen. Differences in the level of plant xylanase inhibitors induced by pathogen were found. Another example is a study on triticale infected by *F. culmorum* where significant increases in β-amylase and α-amylase inhibitor from triticale were found. Data on alterations in protein relative abundance are accompanied by determination of α-amylase and β-amylase enzymatic activities leading to the hypothesis that the activity of plant β-amylase as well as high levels of plant α-amylase inhibitor involved in an inhibition of pathogen α-amylase activity represent important components of plant-pathogen interaction system at molecular level (Perlikowski et al., 2016).

Summary: Protein isoforms and PTMs differ in their primary sequence or amino acid residues modifications, respectively, making them distinguishable on 2DE gels. Different protein isoforms and PTMs derived from a single gene can reveal same, similar, or entirely different biological functions depending on their cellular localizations and protein-protein interactions. Interactomics studies are necessary to understand protein biological functions in the given molecular context.

## Concluding remarks

**Figure 4:**
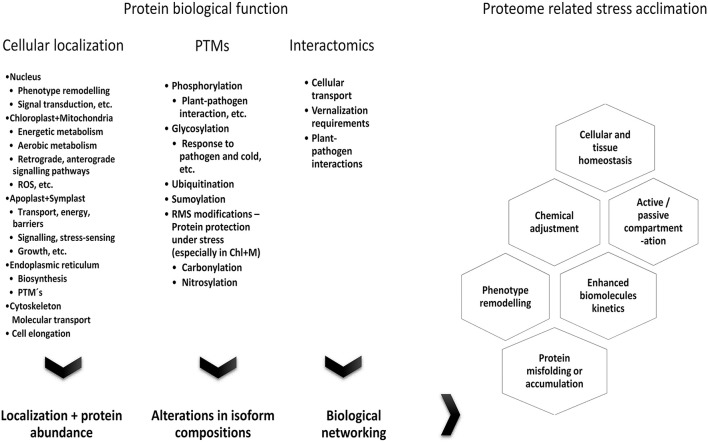
Summary of the major factors determining proteome composition and protein biological functions in stress-treated plants.

Proteins play a crucial role in plant stress response leading to stress-adapted phenotype. However, it is becoming evident that protein role in plant stress response is determined by several factors which include the impacts of environmental stress treatments, genotypic differences affecting stress impacts on plant cellular homeostasis as well as differential protein biological functions determined by protein isoforms, PTMs, cellular localizations, and protein-protein interactions. The major points on the stress-related factors determining protein accumulation and biological function which have to be considered when interpreting proteomic data are summarized below as well as in Figure 4.

- Abiotic stresses reveal both common and specific effects on plants. Several abiotic stresses lead to oxidative stress, several stresses (frost, heat, drought, salinity) lead to cellular dehydration.
- Plant stress response is a dynamic process where several phases (alarm, acclimation, resistance, exhaustion, recovery) with specific proteome composition can be distinguished.
- The impacts of combined stress treatments do not equal to the sum of the effects of the individual stress treatments and thus they need to be studied.

Comparison of stress-susceptible vs. stress-tolerant plants:

- The impacts of stress on plants have to be assessed using plant-related characteristics (e.g., water content in plant tissues) rather than environment-related characteristics (e.g., water content in soil).
- Tolerant plants reveal constitutively enhanced levels of several stress- and detoxification-related proteins thus the stress treatment causes weaker disturbances in their cellular homeostasis.
- Tolerant plants can adjust energy metabolism to enhanced demands of stress acclimation process due to activation of photosynthesis and aerobic respiration.
- Susceptible and tolerant plants also reveal differences in development-related proteins under stress: at molecular level, susceptible plants reveal cellular damage leading to activation of PCD-related processes while tolerant plants reveal restoration of active growth and development.

Protein isoforms and PTMs:

- Protein isoforms reveal differences in their primary sequence while protein PTMs represent differential modifications of amino acid residues.
- Protein isoforms can reveal same, similar or completely different biological functions which depend on their cell localization, PTMs or protein-protein interactions.
- Interactomics studies are necessary to understand protein biological functions in the given molecular context.

## Author contributions

KK outlined the manuscript area and prepared preliminary manuscript text. The other authors PV, MU, IP, and JR contributed actively to the preliminary manuscript text, and designed figures and tables.

### Conflict of interest statement

The authors declare that the research was conducted in the absence of any commercial or financial relationships that could be construed as a potential conflict of interest.

## Abbreviations

ADH: alcohol dehydrogenase
AOX: alternative oxidase
APX: ascorbate peroxidase
AQP: aquaporin
CCOMT: caffeoyl-coenzyme A O-methyltransferase
CBF: C-repeat binding factor
CDPK: calcium-dependent protein kinase
COMT: caffeic acid O-metyltransferase
COR: cold-regulated (protein)
ECM: extracellular matrix
eIF: eukaryotic translation initiation factor
ENO: enolase
ETC: electrontransport chain
FBP ALDO: fructose 1,6-bisphosphate aldolase
GAPDH: glyceraldehyde-3-phosphate dehydrogenase
GLP: germin-like protein
GRP: glycine-rich protein
HDAC: histone deacetylase
HSF: heat-shock transcription factor
HSP: heat-shock protein
LEA: late embryogenesis abundant (protein)
MAPK: mitogen-activated protein kinase
MDAR: monodehydroascorbate reductase
MDH: malate dehydrogenase
MGDG: monogalactosyldiacylglycerol
NDPK: nucleoside diphosphate kinase
OEC: oxygen evolving complex
PCD: programmed cell death
PGK: phosphoglycerokinase
PPR: pentatricopeptide repeat protein
PRK: phosphoribulokinase
Prx: peroxiredoxin
PTM: posttranslational modification
RCS: reactive carbonyl species
RMS: reactive molecular species
RNS: reactive nitrogen species
ROS: reactive oxygen species
RSS: reactive sulfur species
RubisCO: ribulose-1,5-bisphosphate carboxylase
SAM: S-adenosylmethionine
SAMS: S-adenosylmethionine synthetase
SAM22: starvation-associated message 22
SOD: superoxide dismutase
SOS: *salt overly sensitive* (gene)
TCA: tricarboxylic acid (cycle)
TCTP: translationally-controlled tumor protein
TPI: triose phosphate isomerase
Trx: thioredoxin
USP: universal stress protein
VDAC: voltage-dependent anion channel.
