# Supplementary material for: Plant Abiotic Stress Proteomics: The Major Factors Determining Alterations in Cellular Proteome
Source: Front Plant Sci. 2018 Feb 8;9:122. doi: 10.3389/fpls.2018.00122 (PMC5810178; doi:10.3389/fpls.2018.00122)
Supplement: Supplementary file 1 [file Table1.DOCX]

Supplementary Table S1. A list of proteomic studies focused on abiotic stress responses in two or more genotypes (related species) with differential stress tolerance (S1A) and in subcellular proteomes (S1B).

Abbreviations: 2Cys-Prx - 2-cysteine peroxiredoxin; 2DE - two-dimensional electrophoresis; 2D-DIGE - two-dimensional differential in-gel electrophoresis; β-CAS - β-cyanoalanine synthase; ABA - abscisic acid; ACP - acyl carrier protein; ADH - alcohol dehydrogenase; AGPase - ADP glucose pyrophosphorylase; AOC - allene oxide cyclase; AOX - alternative oxidase; APX - ascorbate peroxidase; AQP - aquaporin; AsA - ascorbic acid; bHLH - basic helix-loop-helix (protein); BN-PAGE - blue-native polyacrylamide gel electrophoresis; CA - carbonic anhydrase; CaM - calmodulin; CCOMT - caffeoyl-coenzyme A O-methyltransferase; CHS - chalcone synthase; COMT - caffeic acid O-methyltransferase; COR - Cold-regulated (protein); CPN - chaperonin; CS - cysteine synthase; CDPK - calcium-dependent protein kinase; CHS - chalcone synthase; DAP - differentially abundant proteins; DH - double haploid (line); DHAR - dehydroascorbate reductase; DON - deoxynivalenol; ENO - enolase; ETC - electron transport chain; FBP ALDO - fructose-1,6-bisphosphate aldolase; FIB - fibrillin; FRK - fructokinase; Frx - ferredoxin; FWC - field water capacity; GAPDH - glyceraldehyde-3-phosphate dehydrogenase; GAPDH B - glyceraldehyde-3-phosphate dehydrogenase B form; GDC - glycine decarboxylase; GDH - glutamate dehydrogenase; GLP - germin-like protein; GPX - glutathione peroxidase; GRP - glycine-rich protein; GS - glutamine synthetase; GST - glutathione S-transferase; HPLC - high performance liquid chromatography; Hsc - heat shock cognate protein; IFR - isoflavone reductase; iTRAQ - isobaric tag for relative and absolute quantification; LC - liquid chromatography; LEA - Late embryogenesis-abundant (protein); LOX - lipoxygenase; LTP - lipid transfer protein; LTQ-FTICR - linear quadruple trap-Fourier transform ion cyclotron resonance; MALDI-TOF/TOF - matrix-assisted laser desorption ionization time-of-flight/time-of-flight (spectrometry); MAPK - mitogen-activated protein kinase; MDAR - monodehydroascorbate reductase; MDH - malate dehydrogenase; MIPS - *myo*-inositol-1-phosphate synthase; MS - mass spectrometry; MSSP2 - monosaccharide sensing protein 2; NADP-ME - NADP malic enzyme; NBS-LRR - nucleotide-binding site leucine-rich repeat protein; NEPHGE - non-equilibrium pH gel electrophoresis; NDPK - nucleoside diphosphate kinase; NIL - near-isogenic line; OEE - oxygen evolving enhancer (protein); PAP - polyphosphate-AMP phosphotransferase; PBS - phosphate buffer saline; PC - plastocyanin; PDI - protein disulfide isomerase; PDX - pyridoxal biosynthesis protein; PEG - polyethylene glycol; PGK - phosphoglycerokinase; PGM - phosphoglyceromutase; POX - peroxidase; PPase - inorganic pyrophosphatase; PPDK - pyruvate phosphate dikinase; PPR - pentatricopeptide repeat (protein); PRK - phosphoribulokinase; Prx - peroxiredoxin; PS - photosystem; PVP - polyvinyl pyrrolidone; qTOF - quadrupole time-of-flight; RCA - RubisCO activase; RubisCO - ribulose-1,5-bisphosphate carboxylase/oxygenase; RubisCO LSU - RubisCO large subunit; RubisCO SSU - RubisCO small subunit; Ru5PK - ribulose-5-phosphate kinase; RWC - relative water content; S - susceptible (genotype); SA - salicylic acid; SAMS - S-adenosylmethionine synthase; SBP - sedoheptulose-1,7-bisphosphatase; SHMT - serine hydroxymethyltransferase; SnRK - sucrose non-fermenting-related protein kinase; SOD - superoxide dismutase; SUS1 - sucrose synthase 1; SWC - soil water content; T - tolerant (genotype); t - genotype less tolerant than T; TCA - trichloroacetic acid; TCTP - translationally controlled tumour protein; TF - transcription factor; TLP - thaumatin-like protein; TPI - triose phosphate isomerase; Trx - thioredoxin; TSI-1 - tomato salt-induced 1 (protein); V-ATPase - vacuolar ATPase; VDAC - voltage-dependent anion channel; VSP - vegetative storage protein; WCS - Wheat Cold-specific (protein); WRAB - Wheat responsive-to-ABA (protein); XET - xyloglucan endo-transglycosylase

Supplementary Table S1A. A list of proteomics studies aimed at comparison of plant genotypes or related species with differential stress tolerance.

| Plant material | Treatment | Methods | Major differentially-abundant proteins (DAP) | Reference |  |
| --- | --- | --- | --- | --- | --- |
| **Low temperature (cold, frost)** | |  | | |  |
| Winter wheats (*Triticum aestivum*) Mironovskaya 808 (T) and Bezostaya 1(t) - leaf | 2 °C (21 d) | 0.1 M Tris-HCl, pH 9; 2DE LC-MS/MS | Up: WCS120, WCS19, COR14a - higher levels in T | Vítámvás et al., 2007 |  |
| Winter wheats Norstar (T) and Azar2 (t) - leaf | 2 °C (0, 14, 28, 42, 56 d) | 2DE MALDI-TOF/TOF | Up: COR/LEA (WCOR14a, WRAB17, WRAB18); Cu/Zn-SOD, 2-2-Cys Prx, GST - higher levels in T | Sarhadi et al., 2010 |  |
| Winter wheats Mironovskaya 808 (T) and Bezostaya 1 (t) - crown | 6 °C (0, 3, 21, 84 d) | TCA/acetone/phenol; 2D-DIGE MALDI-TOF/TOF | 298 DAP (202 identified)  Up: 3-PGK, TPI, PGM, ENO; HSP70; MDAR, DHAR, GPX, GST  Down: ALDO, GAPDH; SUS1, UDP-glucose pyrophosphorylase; 14-3-3; HSP90; APX  Vernalization: Chopper chaperone  Genotypic differences: MDH, legumin-like protein - higher in T than t | Vítámvás et al., 2012 |  |
| Winter wheat Samanta (T), spring wheat Sandra (S) - crown | 4 °C (0, 3, 21 d) | TCA/acetone/phenol; 2D-DIGE MALDI-TOF/TOF | 58 DAP (36 identified)  Up: GAPDH, β subunit ATP synthase, CPN60-α, CPN60-β  Down: FRK-2, SUS1, 11S seed storage protein  Genotypic differences: methionine synthase, eIF3, eIF5A2 - higher in T; VER2, sGRP - higher in S | Kosová et al., 2013 |  |
| Winter wheats - Shixin 828 (T), Shiluan 02-1 (t) - leaf | -8 °C (5 h) | TCA/acetone; 2DE MALDI-TOF/TOF | RubisCO LSU and SSU; α and β subunit ATP synthase; V-ATPase; MDH;  Genotypic differences: RubisCO LSU and SSU, PRK; Mn-SOD - higher in T than t | Xu et al., 2013 |  |
| Sunflower (*Helianthus annuus*) cvs Hopi (S), PI543006, BSD-2-691 (T) - leaf | 15/5 °C (day/night) - 7 d | 2DE LC-MS/MS, spectral counting | Identified proteins: 718 (Hopi), 675 (PI543006), 769 (BSD-2-691)  (lipocalin, Trx, 60S ribosomal protein L10A, L4-2, chloroplast 30S ribosomal protein S4) | Balbuena et al., 2011 |  |
| Red clover (*Trifolium pratense*) cv. Endure and Christie | Natural hardening in Québec, Canada: control (October), cold acclimation (January, February) | 2D-DIGE MALDI-TOF/TOF | Up: VSP, dehydrins  Down: Prx (Christie), PR1 (Endure)  Genotypic differences: eIF5A-like (Christie); eEF1γ, eEF2-like; peptidase M1 family aminopeptidase (Endure); | Bertrand et al., 2016 |  |
| **Heat** |  |  |  |  |  |
| Common wheat - Fang (T), Wyuna (S) - grain endosperm | 40/25 °C (day/night) - 15,16,17 d post-anthesis | TCA/acetone 2DE MALDI-TOF; MS/MS Q-TOF | Genotypic differences: Seven small HSP (16.9 kD class I HSP) proteins unique to T | Skylas et al., 2002 |  |
| **Drought** |  |  |  |  |  |
| Common wheat - spring wheats Arvand, Khazar-1, Kelk Afghani - grain | Field conditions (Azarbayjan) plus artificial irrigation | 2DE MALDI-TOF/TOF | 121 (57 identified)  Up: Trx *h*, 1-Cys peroxiredoxin, GST; PDI; LEA, sHSP17, HSP70 | Hajheidari et al., 2007 |  |
| Wheat Iranian landraces N14, N49- stem (senescence stage) | 80-90% FWC (control), 50% FWC (drought) - 10, 20, 30 days after anthesis | 2DE MALDI-TOF/TOF; Q-TOF | 135 DAP (82 identified) - redox (GST, Prx-5, Trx M), chaperonin, protein metabolism (chloroplast 50S ribosomal protein L4, proteasome regulatory subunit 14, Clp protease) | Bazargani et al., 2011 |  |
| Australian wheats Kukri (S), Excalibur, RAC875 (T) - leaf | Water withholding until leaf wilting in Kukri (S) - 14, 24 d, and rewatering (25 d) | TCA; nanoLC-MS/MS iTRAQ 8plex | 1299 identified proteins  Increase in ROS metabolism-associated proteins (CAT, Cu/Zn-SOD, Mn-SOD), decrease in photosynthesis and Calvin cycle-related proteins (RubisCO; PSI subunit VII PsaC)  Genotypic differences: COR410 - higher increase in T than S | Ford et al., 2011 |  |
| Wheat spring genotypes Ningchun 4 (T), Chinese Spring (S) - grain | 14 days after flowering | 2DE MALDI-TOF, MALDI-TOF/TOF | 152 DAP (96 identified) - Up: TCTP, Down: APX, RubisCO, | Ge et al., 2012 |  |
| Common wheat cv. Nesser (T), Opata M85 (S) - root | 21 °C; 40 % humidity - combined effect of drought and ABA (100 µM) | nanoLC-MS/MS iTRAQ | 1656 identified proteins  805 ABA-responsive proteins: LEA, protein phosphatases PP2C;  Genotypic differences: HSP70, HSP90; 14-3-3, G-proteins; V-ATPase - higher in T; β-expansin, porins - higher in S | Alvarez et al., 2014 |  |
| Wheat cvs. Seri M82 (T), SW89.5193 (S) - leaf, root | 80% FWC (control), 20% FWC (drought) | 2DE nanoLC-ESI-MS/MS | Up: GST, SOD  Genotypic differences: MDH, SAMS, Frx NADP reductase - up in T; 14-3-3 - down in S | Faghani et al., 2015 |  |
| Wheat cvs Xihan 2 (T), Longchun 23 (S) - leaf | 18, 24, 48 h water withholding; 24 h rehydration | 2DE MALDI-TOF/TOF MS | 84 (T), 64 (S) DAP. Up: Osmolyte biosynthesis: GS (T), SAMS 2 (S); ATP synthase, GST, GPX (T)  Down: Slight decrease OEE1-2 (T); large decrease OEE2 (S) | Cheng et al., 2016 |  |
| Durum wheat cv. Kiziltan (S), emmer (*T. dicoccoides*) lines TR39477, TTD22 (T) - leaf | 9 d water withholdinging | 2DE nanoLC-ESI-MS/MS | 75 identified proteins, 11 candidates for drought tolerance  Genotypic differences: TPI, ATP synthase CF1 (efficient carbohydrate metabolism and ATP production) - higher in T; β-1,3-glucanase, β-1,4-glucanase, XET (cell wall remodelling for osmotic adjustment and energy source); methionine synthase - higher in S | Budak et al., 2013 |  |
| Barley cv. Basrah (T) and Golden Promise (S) - leaf, root | 7 d water withholdinging  Control: 80 % RWC; Drought: 70 % RWC (T), 60 % RWC (S) | 10 mM PBS, TCA-acetone; 2D-DIGE MALDI-TOF | Identified proteins: 24 (leaf), 45 (root)  Up: ABA-induced protein r40c1, small G-protein Rab2, Myb-like protein, 14-3-3 protein  Down: GST, GPX  Genotypic differences: Enhanced regulation of ROS (APX, CAT, LOX, class III POX) and protein folding in T than in S | Wendelboe-Nelson and Morris, 2012 |  |
| Barley - 8 Egyptian accessions, 2 selected for proteome analysis 15141 (T), 15163 (S) - leaf | 24 °C; 70 % FWC (control); 5 days at 10 % FWC (stress) | TCA/acetone; 2D-DIGE MALDI-TOF | Up: PDI, Hsp90, Hsp100 (Clp protease), chloroplastic ATP synthase CF1 α;  Genotypic differences: PPDK, Hsp70, zinc metalloprotease - higher in T than S; proteins involved in osmolyte biosynthesis (betaine aldehyde dehydrogenase, methionine synthase, SUS1) - higher in S than T | Ashoub et al., 2013 |  |
| Barley lines 004186 (S), 004223 (T) - shoot | 20% PEG; 3 d water withholding | 2DE MALDI-TOF, nanoLC-MS/MS | Up: V-ATPase, MDH, OEE (T), HSP70 - up (T), down (S)  Down: RubisCO activase (S) | Kausar et al., 2013 |  |
| Maize (*Zea mays*) lines F2, *Io* - leaf | 0, 14 d water withholding | 2DE Edman sequencing | Up: cystatin, COMT, ENO, MDH, PPase, β-1,3-glucosidase | Riccardi et al., 2004 |  |
| Maize lines F2, *Io* - leaf |  | 2DE Edman sequencing, nano LC ESI-MS/MS | Up: CCOMT, COMT, methionine synthase, SAMS | Vincent et al., 2005 |  |
| Maize line FR697 - root elongation zone - cell wall (apoplast) | -0.03 MPa (control); -1.6 MPa (dehydration) | 2DE HPLC-ESI-Q-TOF MS | Cell wall apoplast proteins: expansin, XET, β-D-glucosidase | Zhu et al., 2007 |  |
| Maize genotypes CE704 (T), 2023 (S) - leaf | 6 d water withholding | 2DE MALDI-TOF, iTRAQ | Several isoforms (HSP26, HSP70, 14-3-3) | Benešová et al., 2012 |  |
| Maize genotypes CE704 (T), 2023 (S), CE704×2023 and 2023×CE704 hybrids - leaf | 10 d of water withholding | 2DE MALDI-TOF, iTRAQ | 2023 (S): Decreased Frx NADP reductase, ATP synthase subunits  Hybrids vs parents: Decreased proteins of photosynthesis ETC: PSII: D1, CP47; cytb_559_α; PsbH, OEE3; chloroplast ribosomal proteins (2023×CE704) | Holá et al., 2017 |  |
| Sugar beet (*Beta vulgaris*) - genotypes 7112, 7219-P6.9 - leaf | 157 d - field; irrigated (control), non-irrigated (drought) | 2DE LC-MS/MS | 157 DAP (20 identified - RubisCO, sHSP, NDPK, redox reulation  Down: α-NAC | Hajheidari et al., 2005 |  |
| Sunflower (*Helianthus annuus*) lines RGK21 (S), BGK329 (T) - | Field experiment - water withholding at flowering stage | 2DE nanoLC-MS/MS | Genotypic differences: APX, Cu/Zn-SOD; ENO, PGM - up in T, down in S; CHS - up in S, down in T | Ghaffari et al. 2013 |  |
| root |  |  |  |  |  |
| Osmotic stress (PEG-6000) | |  |  |  |  |
| Common wheat - spring wheats Abbondanza (T), Qingchun 38 (S) - leaf | PEG-6000 (-1 MPa; 72 h), recovery (24 h) | TCA/acetone; 2DE MALDI-TOF/TOF | 38 (35 identified proteins)  Up: GAPDH B; 26S proteasome, V-ATPase A  Down: RubisCO LSU and SSU, GAPDH, TPI, AGPase (starch biosynthesis)  Genotypic differences: Less PEG-affected proteins in T than S | Ye et al., 2013 |  |
| Common wheat cv Hanxuan 10 (T) and Ningchun 47 (t) - seedling leaf phosphoproteome | Hoagland solution, 20% PEG-6000  (-075 MPa) for 48 h | TCA/acetone/phenol; phosphopeptide enrichment via TiO_2_ microcolumns; LC-MS/MS | 173 (T) and 251 (t) phosphoproteins identified  Phosphoproteins identified: signalling (SnRK2 kinase, protein phosphatase 2C, CDPK, calmodulin 2-2); transport (AQP, MSSP2; H^+^-ATPase); LEA proteins (WCOR719, WCOR825, WRAB17) | Zhang et al., 2014 |  |
| Common wheat cv Ningchun 47 (T), Chinese Spring (S) - leaf | PEG-6000 | 2DE MALDI-TOF/TOF | 146 DAP  Genotypic differences: ENO, GAPDH, OEE2, fibrillin-like, 2-Cys Prx BAS1, HSP70, CPN60α, 50S ribosomal protein L1 (up in Ningchun) | Cheng et al., 2015 |  |
| **Waterlogging** |  |  |  |  |  |
| Maize (*Zea mays*) A3237 (T), A3239 (S) - seedling root | 7 d waterlogging | iTRAQ LC-MS/MS | 3318 identified; 211 DAP - Up: ADH, NADP-ME, XET6, GST, | Yu et al., 2015 |  |
| **Salinity** |  |  |  |  |  |
| Common wheat (*T. aestivum*) Jinan 177 (S), *T. aestivum* × *Thinopyrum ponticum* Shanrong 3 (T) - seedling root | ½ Hoagland solution, 200 mM NaCl (24 h) | TCA/acetone; 2DE MALDI-TOF, MALDI-TOF/TOF | 114 (110 identified - 49 salt-responsive, 34 genotypic differences)  Up: 14-3-3;  Down: tubulin α-3  Genotypic differences: DEAD-box RNA helicase, DWARF3 (GA biosynthesis), eIF5A2, V-ATPase subunit E - higher in T; G-protein β subunit, ethylene receptor ETR1 - higher in S | Wang et al., 2008 |  |
| Common wheat cv. Calingiri, Janz (S), Wyalkatchem (T) - shoot mitochondrial fraction | 200 mM NaCl (increase per 50 mM NaCl/d) | Isolation: PVP gradient; acetone extraction; 2D-DIGE LC-MS/MS | 192 DAP (68 identified)  Up: AOX, Mn-SOD, VDAC  Down: CS, NDPK, outer mitochndrial membrane porin  Genotypic differences: AOX, Mn-SOD - higher in T | Jacoby et al., 2010 |  |
| Common wheat cvs. Jing-411 (T), Chinese Spring (S) - seedling root | 0.5, 1.5, 2.5% NaCl (2 d) | 2DE MALDI-TOF/TOF | 198 DAP (144 identified). Genotypic differences: RubisCO LSU binding protein CPN60-α, PR10 - higher in T than S | Guo et al., 2012 |  |
| Common wheat (*T. aestivum*) cv Chinese Spring (S), *T. aestivum* × *Lophopyrum elongatum* amphiploid (T) - mitochondrial fraction (shoot, root) | 200 mM NaCl (increase per 50 mM NaCl/d) | 100% acetone (leaf), TCA/acetone (root); 2D-DIGE MALDI-TOF/TOF; HPLC Q-TOF MS/MS (peptide fingerprinting - genotypic differences) | 55 root, 15 shoot differentially abundant proteins  Organ-specific differences: aspartate aminotransferase, GDH (up in shoot, down in root)  Genotypic differences: Mn-SOD, MDH, aconitase, SHMT, β-CAS - higher in T | Jacoby et al., 2013 |  |
| Barley cv OUK305 (T), OUI743 (S) - root | 200 mM NaCl (5 d) | 40 mM Tris, 8 M urea, 4% CHAPS, 0.2% Bio-Lyte; 2DE nanoLC-ESI-MS/MS | 6 differentially abundant proteins CCOMT, DHAR, GST (2 spots), POX, PR10 - higher in T than S | Sugimoto and Takeda, 2009 |  |
| Barley cv Morex (T), Steptoe (S) - root | 100, 150 mM NaCl (13 d) | TCA/acetone; 2DE MALDI-TOF; nanoLC-ESI-Q-TOF MS/MS | 39 differentially abundant proteins  Up: LOX1, POX, SAMS, β-1,3-glucanase  Down: IDI1, IDI2, IDS2, IDS3,  Genotypic differences: class III POX, SAMS - higher in T; APX, MDAR - higher in S | Witzel et al., 2009 |  |
| Barley cv Afzal (T), L-527 (S) - leaf | 300 mM NaCl (increase per 50 mM NaCl/d) 24 h | TCA/acetone; 2DE MALDI-TOF/TOF | 117 DAP (22 identified proteins)  Up:, PC, OEE2, PSI subunit VII (PsaC), PRK; 2-Cys Prx, Trx, GST, SOD; TPI, FBP ALDO - higher in T than S | Rasoulnia et al, 2011 |  |
| Barley cv Afzal (T), L-527 (S) - leaf | 300 mM NaCl (increase per 50 mM NaCl/d) - 21 d | TCA/acetone; 2DE MALDI-TOF/TOF | 44 DAP  Up (43): RubisCO LSU, SSU, RubisCO activase, OEE2; NDPK; GLP; profilin; ribosomal protein L12, 30S ribosomal protein S1; translationally-controlled tumor protein homolog  Genotypic differences: DHAR, Trx - higher in S | Fatehi et al., 2012 |  |
| Barley cv Morex (T), Steptoe (S) - root | 100, 150 mM NaCl (0, 1, 4, 7, 10 d) | TCA/acetone; 2DE MALDI-TOF; nanoLC-ESI-Q-TOF MS/MS | 91 DAP (74 identified proteins)  Genotypic differences: GLP3-7, GLP12, β-1,3-glucanase, ATP synthase CF1 β - higher in T; GLP5a, PR17 - higher in S | Witzel et al., 2014 |  |
| Barley lines DH14 (S), DH187 (T) - root | 100 mM NaCl (6 d) | 2DE MALDI-TOF/TOF | Genotypic differences: annexin, TCTP, LOX, osmotin, V-ATPase - higher in T than S | Mostek et al., 2015 |  |
| Canola (*Brassica napus*) cvs Hyola 308 (T), Sarigol (S) - leaf | 0, 175, 350 mM NaCl | 2DE MALDI-TOF/TOF | 44 DAP (T), 33 DAP (S) (Cu/Zn-SOD, PRK; PSBO2, ATP synthase CF1γ, 30S ribosomal protein S10; RubisCO LSU, SSU) | Bandehagh et al., 2011 |  |
| Potato (*Solanum tuberosum*) cvs Concord (S), Kennebec-(T) - shoot | 90 mM NaCl | 2DE Edman sequencing | 47 DAP: Up: calreticulin, TSI-1, osmotin-like, HSP  Down: RubisCO, carbonic anhydrase, OEE; GS - more in S than T | Aghaei et al., 2008 |  |
| Tomato (*Lycopersicum esculentum*) - cvs Cervil, Super Marmande (S), Levovil, Roma (T) - root | 100 mM NaCl (14 d) | 2DE nanoLC-MS/MS | 90 DAP; Up: HSP90; Down: V-ATPase  Genotypic differences: Trx-H - increased in S and decreased in T  TSI-1, PR10 - in all genotypes, highest in Super Marmande | Manaa et al., 2011 |  |
| Cowpea (*Vigna unguiculata*) cv. Pitiúba (T), TVu 2331 (S) - leaf | 75 mM NaCl | 2DE LC-ESI-MS/MS | Genotypic differences:RCA, Ru5PK, OEE2, glycine decarboxylase - increased in T; OEE1, Mn-stabilizing protein II, CA, RubisCO - decreased in S | de Abreu et al., 2014 |  |
| Rice (*Oryza sativa*) - IR64 (S), IR64 mutants 167-1-3, S-730-1 (T) - seedling shoot | 12 dS m^-1^ (NaCl) | 2DE MALDI-TOF/TOF | 854 proteins, 67 DAP | Ghaffari et al., 2014 |  |
| Imbalances in mineral nutrients | |  |  |  |  |
| Boron |  |  |  |  |  |
| Barley Clipper (S) × Sahara (T) DH lines - leaf, root | 1 mM H_3_BO_3_ (S), 5 mM H_3_BO_3_ (T) for 14 d | PBS pH 7.5, TCA/acetone; 2D-nanoLC-MS/MS iTRAQ | 138 (leaf), 341 (root) identified proteins  Up: IDS2, IDS3, methyl-thioribose kinase  Leaf: PRK, PGK, PGM, ENO, PC, RubisCO activase, eEF1-α,β,γ; eEF-G, eEF-Tu; TLP; Cu/Zn-SOD; 50S ribosomal protein L3; 60S ribosomal protein L1  Root: CCOMT, class III POX, chitinase, 26S proteasome , β-1,3-glucanase; ATP synthase CF1 β, IDS2, IDS3; Hsp70; Hsc70; 40S ribosomal protein S5 | Patterson et al., 2007 |  |
| Flax (*Linum usitatissimum*) - cvs. Jitka, Tábor - hypocotyl cell suspension culture | 10, 50, 100 µM Cd(NO_3_)_2_ for 48 h | 2DE MALDI-TOF/TOF | 14 DAP (ferritin, GS, lipocalin-1, α-tubulin 3, SAMS, HSP70, HSP83; IFR) | Hradilová et al., 2010 |  |
| Combined stress |  |  |  |  |  |
| Osmotic stress or salinity - common wheat (*T. aestivum*) Jinan 177 (S), *T. aestivum* × *Thinopyrum ponticum* Shanrong 3 (T) - root, | ½ Hoagland solution  18% PEG-6000 or  200 mM NaCl (24 h) | TCA/acetone; 2DE MALDI-TOF/TOF | 93 (root), 65 (leaf) differentially abundant proteins; 34 (root), 6 (leaf) - genotypic differences  PEG: 38 root, 39 leaf; Salinity: 52 root, 52 leaf proteins  PEG-specific proteins: ribosomal protein S8 (↓)  Salt-specific proteins: importin α 1b (root),  Genotypic differences: chl *a*/*b* binding apoprotein CP24 precursor, DWARF3 - higher in T | Peng et al., 2009 |  |
| leaf |  |  |  |  |  |
| Drought and heat  Barley - Syrian landrace Arta (T), Australian cv Keel (T) - leaf (heading stage) | Drought: 50% FWC (control), 15% FWC (stress) for 3d  Heat: 36 °C (4 h) | TCA/acetone; 2DE, 2D-DIGE MALDI-TOF/TOF | 99 DAP  Heat - up: FBP ALDO, chaperones, proteases, eEF-G, eIF4A, RubisCO activase B  Genotypic differences (14 proteins): photosynthesis-related proteins (LHCII type III Lhcb3, OEE1 PsbO, RubisCO activase B) - higher in Keel than Arta | Rollins et al., 2013 |  |
| Drought, salinity - grapevine (*Vitis vinifera*) - cvs Chardonnay, Cabernet Sauvignon - shoot | Drought:water withholding  Salt: gradual increase 0 to 250 mM NaCl in 16 d | 2DE MALDI-TOF/TOF | 191 identified proteins - Up: PR10, Prx  Genotypic differences: PPR | Vincent et al., 2007 |  |
| Chickpea (*Cicer arietinum*) - cvs ICCV-2 (S), JG-62 (T) - nuclear fraction | Drought: water withholding 24-144 h | Nuclear fraction: 2DE ESI-MS/MS | 75 DAP  Genotypic differences: H2B, H3 - up in T, down in S  DNA cytosine methyltransferase Zmet3 - up in T;  ROS scavenging - APX, SOD, GPX - up in T | Subba et al, 2013 |  |
| *Festuca pratensis* - S, T | Cold |  | Genotypic differences: RubisCO activase - higher in T than S | Kosmala et al., 2009 |  |
| *Festuca arundinacea* - T, S chloroplast fraction | Drought | 2-DE; nano-UPLC MS/MS | Drought: Up: RubisCO activase; Down: lipocalin  Genotypic differences: OEE2, lipocalin - up in T; | Kosmala et al., 2012 |  |
| Oilseed rape (*Brassica napus*) leaf - Californium (C), Cadeli (D) - water savers; Navajo (N), Viking (V) - water spenders | Drought | 2D-DIGE, MALDI-TOF/TOF | Water savers (C+D): Up: nitrogen assimilation, ATP, redox homeostasis;  Water spenders (N+V): carbohydrate and energy metabolism, photosynthesis, stress-related, rRNA processing | Urban et al., 2017 |  |
| Related species with contrasting stress tolerance (glycophyte vs halophyte) | | | |  |  |
|  | | | |  |  |
| Rice (*Oryza sativa*) (S) vs *Porteresia coarctata* (T) - leaf | 200, 400 mM NaCl (72 h) | 2DE MALDI-TOF | *P. coarctata*: higher levels of photosynthesis-related proteins (33 kDa Mn-stabilizing OEC, CP47, RubisCO, RubisCO activase); HSP70; cellulose synthase; *myo*-inositol-1-phosphate synthase (INPS), energy-saving enzymes sucrose synthase (SUS) than in *O. sativa* | Sengupta and Majumder, 2009 |  |
| *Arabidopsis thaliana*  (S) vs *Thellungiella halophila* (T) leaf | 50, 150 mM (5 d) | 2DE MALDI-TOF/TOF; iTRAQ LC-MS/MS | *Arabidopsis*: 88 DAPs, 79 identified; upregulation of respiratory enzymes; JA metabolism (AOC2, LOX2), ion transport (V-ATPase), ROS and defense (GST, PR5); downregulation of RubisCO activase, ribosomal proteins (S5, L29)  *Thellungiella*: 37 differentially abundant proteins, 32 identified; Up: RubisCO activase; ROS (APX), ribosomal proteins (S7, S15A, S24); HSC70-3, P5CS, FtsH protease, eIF3A | Pang et al., 2010 |  |
| *Hordeum marinum* (T) vs *Hordeum vulgare* cv Tadmor (S) crown | 300 mM NaCl (5 d) | 1DE nanoLC-ESI-MS/MS | Genotypic differences: NACα; OEE1,2; different isoforms: eIF5A1 (*H. vulgare*) vs eIF5A2 (*H. marinum*); TCTP - PCD inhibition (higher in *H. marinum*); FAS-associated factor 2B - PCD induction (present in *H. vulgare* only); 60S acidic ribosomal protein P2 (lower in *H. vulgare* than *H. marinum*); 26S protease 6A (higher in *H. vulgare* than *H. marinum*) | Maršálová et al., 2016 |  |
| Supplemental Table S1B. A list of abiotic stress-related organellar proteomics studies. | | | | |  |
| Plant material | Treatment | Methods | Major differentially-abundant proteins (DAP) | Reference |  |
| **Cell wall** |  |  |  |  |  |
| Rice | Drought | 2DE | 192 DAP; signalling: 14-3-3; inositol phosphatase; GF14-b, GF14-c; 20S proteasome α; oligopeptidase A-like; ALDO, ENO, PRK; SAM 2-demethylmenaquinone methyltransferase-like; ADK, AdoHcyase; Trx h, Trx m, 2-Cys Prx, MDAR; DnaK chaperone | Pandey et al., 2010 |  |
| Soybean (*Glycine max*) cv. Enrei - root cell wall | 2d waterlogging |  | 204 identified cell wall proteins - 16 DAP Down: GLP, LOX, stem glycoprotein, Cu/Zn-SOD; pI change in copper amino oxidase | Komatsu et al., 2010 |  |
| Chickpea (*Cicer arietinum*) - JG-62 (T) | Drought | 2DE | APX, Trx m, mannose lectin, glyoxalase I, GRPs, NDPK, receptor-like kinase CHRK1, protein kinase 2; cell wall modification: cellulose synthase-like, glucan *endo*-1,3-β-D-glucosidase; Met synthase | Bhushan et al., 2007 |  |
| Maize line FR697 - root elongation zone - cell wall (apoplast) | -0.03 MPa (control); -1.6 MPa (dehydration) | 2DE HPLC-ESI-Q-TOF MS | Cell wall apoplast proteins: expansin, XET, β-D-glucosidase | Zhu et al., 2007 |  |
| **Plasma membrane** | |  |  |  |  |
| Soybean (*Glycine max*) cv Enrei - seedling root and hypocotyl plasma membrane fraction | Flooding | 2DE MS; nanoLC-MS/MS | Signalling: 14-3-3, Ser/Thr protein kinase; Hsc-70; SOD; ATP synthase CF1α | Komatsu et al., 2009 |  |
| Soybean (*Glycine max*) cv Enrei - seedling root and hypocotyl plasma membrane fraction | 10% PEG-6000 (2 d) | 2DE, nanoLC-MS/MS | Up: calnexin, H^+^-ATPase, ribosomal protein S10, TPI | Nouri and Koamtsu, 2010 |  |
| Barley - seed aleurone layer |  |  |  | Hynek et al., 2009 |  |
| **Nucleus** |  |  |  |  |  |
| *Arabidopsis thaliana* | Cold |  | Up: HSP70, HSP90-like, dnaK type chaperone Hsc70-1; TFs: AtMYB2, MYB34, bZIP TF OBF4, bHLH TF MYC; DNA- and RNA-associated proteins: U2 sn ribonucleoprotein A, helicase C in DEAD/DEAH box, CHP-rich Zn-finger protein, DNA-damage-repairř protein DRT102; ribosomal proteins: 60S acidic ribosomal proteins P0, P2-A, L12  Down: calmodulin, GLP, HSF8, 20S proteasome α | Bae et al., 2003 |  |
| Chickpea (*Cicer arietinum*) | Drought |  | Signalling: 14-3-3, Ser-Thr kinase, His kinase, GAPDH; AP2/EREBP, homeobox leucine-zipper; GRP1, GRP2; DnaJ, dehydrin homolog Wcs66; H2b, H3, DNA cytosine methyltransferase Zmet3, HDAC; RanBP | Pandey et al., 2008 |  |
| Rice (*Oryza sativa*) | Drought |  | Up: GLP, Cu/Zn-SOD, 2-Cys Prx, PR10a, chitinase, endo-1,3-glucanase; ribosomal proteins: 50S L-22, S6 (↑), L12, 60S P2A (↓) | Choudhary et al., 2009 |  |
| *Xerophyta viscosa* | Drought |  | 438 protein spots: 18 up: Zn-finger helicase TF; gene regulation: non-LTR retrotransposons, maturases; chaperonin; EF-Tu precursor, ribosomal L28 | Abdalla et al., 2010 |  |
| Chickpea (*Cicer arietinum*) - cvs ICCV-2 (S), JG-62 (T) - nuclear fraction | Drought: water withholding 24-144 h | Nuclear fraction: 2DE ESI-MS/MS | 75 DAP  Genotypic differences: H2B, H3 - up in T, down in S  DNA cytosine methyltransferase Zmet3 - up in T;  ROS scavenging - APX, SOD, GPX - up in T | Subba et al., 2013 |  |
| Soybean cv. Enrei - root tip - nuclear fraction | Flooding | Nuclear fraction: | Up:  Down: RACK1, splicing factor PWI domain-containing, epsilon2-COP, beta catenin, clathrin heavy chain | Komatsu et al., 2014 |  |
| Soybean - phosphoproteome | Flooding |  |  | Yin and Komatsu, 2015 |  |
| Soybean | Flooding |  | 365 nuclear DAP: Down: H1, H3; NOP1/NOP56; 60S preribosome; pre-mRNA processing (spliceosome components) | Yin and Komatsu, 2016 |  |
| **Mitochondria** |  |  |  |  |  |
| Pea (*Pisum sativum*) cv. Green Feast - leaf | Cold (4 °C; 36 h)  Drought (7 d waterwithholding)  Paraquat (662.5 mg/L) | 2DE Q-TOF MS; BN-PAGE | Up: GDC, SHMT isoforms; MDH, ATP synthase α,β,γ; HSP22, HSP70, HSP90, chaperonin 10; βCA | Taylor et al., 2005 |  |
| Common wheat cv. Calingiri, Janz (S), Wyalkatchem (T) - shoot mitochondrial fraction | 200 mM NaCl (increase per 50 mM NaCl/d) | Isolation: PVP gradient; acetone extraction; 2D-DIGE LC-MS/MS | 192 DAP (68 identified)  Up: AOX, Mn-SOD, VDAC  Down: CS, NDPK, outer mitochndrial membrane porin  Genotypic differences: AOX, Mn-SOD - higher in T | Jacoby et al., 2010 |  |
| Common wheat (*T. aestivum*) cv Chinese Spring (S), *T. aestivum* × *Lophopyrum elongatum* amphiploid (T) - mitochondrial fraction (shoot, root) | 200 mM NaCl (increase per 50 mM NaCl/d) | 100% acetone (leaf), TCA/acetone (root); 2D-DIGE MALDI-TOF/TOF; HPLC Q-TOF MS/MS (peptide fingerprinting - genotypic differences) | 55 root, 15 shoot differentially abundant proteins  Organ-specific differences: aspartate aminotransferase, GDH (up in shoot, down in root)  Genotypic differences: Mn-SOD, MDH, aconitase, SHMT, β-CAS - higher in T | Jacoby et al., 2013 |  |
| Soybean - root, hypocotyl | Flooding |  | Up: TCA cycle enzymes, NADH-ubiquinone oxidoreductase; GroES chaperonin, EF-Tu, porin, VDAC;  Down: cyt c, Tim, Tom20; protein components of complexes III, IV and V of ETC | Komatsu et al., 2011 |  |
| Pea cv. Lincoln - S-nitrosylation | 150 mM NaCl (5, 14 d) |  | SNO modified proteins: ATP synthase β, HSP90, SHMT; Prx IIF - SNO - decreased activity | Camejo et al., 2013 |  |
| **Chloroplasts** |  |  |  |  |  |
| *Arabidopsis thaliana* - chloroplast lumen and stromal proteome | Cold (5 °C; 1, 10, 40 d) | 2D-DIGE | 43 DAP: Up: AOC2; SHMT; FIB; CAT-2, glyoxalase I, Trx m, PGM; ATP synthase α,β,γ,δ  Down: RubisCO activase (RCA), 2-Cys PrxA,B; CA-1; PGK, PRK, quinone oxidoreductase | Goulas et al., 2006 |  |
| Common wheat cv. Keumgang - leaf chloroplast fraction | 150 mM NaCl  (1, 2, 3 d) | Isolation: Percoll gradient; TCA/acetone; 2DE LTQ-FTICR-MS | 100 DAP (65 identified)  Up: RubisCO, cyt b6-f, GAPDH, GDH, GLP, GS, PDX1.2, PDX1.3  Down: ATP synthase α,β,γ; V-ATPase | Kamal et al., 2012 |  |
| *Festuca arundinacea* - T, S | Drought | 2-DE; nano-UPLC MS/MS | Drought: Up: RubisCO activase; Down: lipocalin  Genotypic differences: OEE2, lipocalin - up in T; | Kosmala et al., 2012 |  |
| Maize (*Zea mays*) - hybrid SR12 (T) - leaf chloroplast fraction | 1, 25 mM NaCl (1, 2, 4 h) | 2DE MALDI-TOF | Up: 12 (ferredoxin-NADP reductase, 23 kDa PSII, FtsH-like; ATP synthase CF1ε);  Down: 8 (ATP synthase CF1α, LHC *a*/*b* binding protein) | Zörb et al., 2009 |  |
| Tomato cv. Crovarese - leaf chloroplast fraction | Drought (19 d), recovery (6 d) | 2D-DIGE nanoLC-ESI-LIT-MS/MS | Down: photosynthesis (PC, chl binding protein4; PGK, PRK), ATP synthase CF1β,γ; Up: APX1, PAP | Tamburino et al., 2017 |  |
| **Endoplasmic reticulum** | |  |  |  |  |
| Soybean | Flooding | 1DE | DAP: 117↑212↓  111 proteins Protein synthesis, folding, degradation, PTMs, e.g., luminal-binding protein 5, arabinogalactan protein 2, methyltransferase PMT2  Fatty acid biosynthesis: 3-ketoacyl-CoA reductase | Komatsu et al., 2012 |  |
| **Tonoplast (Vacuole)** | |  |  |  |  |
| *Mesembryanthemum crystallinum* | Salinity | 2D-DIGE | V-ATPase, ALDO, ENO | Barkla et al., 2009 |  |
| Additional references in Supplementary Tables S1A and S1B which are not cited in the manuscript text:  Abdalla, K.O., Baker, B., and Rafudeen, M.S. (2010). Proteomic analysis of nuclear proteins during dehydration of the resurrection plant *Xerophyta viscosa*. *Plant Growth Regul.* 62, 279–292. doi: 10.1007/s10725-010-9497-2  Aghaei, K., Ehsanpour, A.A., and Komatsu, S. (2008). Proteome analysis of potato under salt stress. *J. Proteome Res.* 7, 4858–4868. doi: 10.1021/pr800460y  Ashoub, A., Beckhaus, T., Berberich, T., Karas, M., and Brüggemann, W. (2013). Comparative analysis of barley leaf proteome as affected by drought stress. *Planta* 237, 771–781. doi: 10.1007/s00425-012-1798-4  Balbuena, T.S., Salas, J.J., Martinez-Force, E., Garces, R., and Thelen, J.J. (2011). Proteome analysis of cold acclimation in sunflower. *J. Proteome Res.* 10, 2330–2346. doi:**:** 10.1021/pr101137q  Bandehagh, A., Salekdeh, G.H., Toorchi, M., Mohammadi, A., and Komatsu, S. (2011). Comparative proteomic analysis of canola leaves under salinity stress. *Proteomics* 11, 1965–1975. doi: 10.1002/pmic.201000564  Bazargani, M.M., Sarhadi, E., Bushehri, A.A.S., Matros, A., Mock, H.P., Naghavi, M.R., Hajihoseini, V., Mardi, M., Hajirezaei, M.R., Moradi, F., *et al.*, (2011). A proteomics view on the role of drought-induced senescence and oxidative stress defense in enhanced stem reserves remobilization in wheat. *J. Proteom.* 74, 1959–1973. doi: 10.1016/j.jprot.2011.05.015  Bertrand, A., Bipfubusa, M., Castonguay, Y., Rocher, S., Szopinska-Morawska, A., Papdopoulos, Y., and Renaut, J. (2016). A proteome analysis of freezing tolerance in red clover (*Trifolium pratense* L.). *BMC Plant Biol.* 16, 65. doi:  [10.1186/s12870-016-0751-2](https://dx.doi.org/10.1186%2Fs12870-016-0751-2)  Cheng, Z.W., Dong, K., Ge, P., Bian, Y.W., Dong, L.W., Dong, X., Li, X.H., and Yan, Y.M. (2015). Identification of leaf proteins differentially accumulated between wheat cultivars distinct in their levels of drought tolerance. *PLoS ONE* 10, e0125302. doi: [10.1371/journal.pone.0125302](https://doi.org/10.1371/journal.pone.0125302)  Choudhary, M.K., Basu, D., Datta, A., Chakraborty, N., and Chakraborty, S. (2009). Dehydration-responsive nuclear proteome of rice (*Oryza sativa* L.) illustrates protein network, novel regulators of cellular adaptation, and evolutionary perspective. *Mol. Cell. Proteomics* 8, 1579–1598. doi: 10.1074/mcp.M800601-MCP200  de Abreu, C.E.B., dos Santos Araújo, G., de Oliveira Monteiro-Moreira, A.C., Costa, J.H., de Brito Leite, H., Moreno, F.B.M.B., Prisco, J.T., and Gomes-Filho, E. (2014). Proteomic analysis of salt stress and recovery in leaves of *Vigna unguiculata* cultivars differing in salt tolerance. *Plant Cell Rep.* 33, 1289–1306. doi: 10.1007/s00299-014-1616-5  Fatehi, F., Hosseinzadeh, A., Alizadeh, H., Brimavandi, T., and Struik, P.C. (2012). The proteome response of salt-resistant and salt-susceptiblebarley genotypes to long-term salinity stress. *Mol. Biol. Rep.* 39, 6387–6397. doi: 10.1007/s11033-012-1460-z  Ghaffari, A., Gharechahi, J., Nakhoda, B., and Salekdeh, G.H. (2014). Physiology and proteome responses of two contrasting rice mutants and their wild type parent under salt stress conditions at the vegetative stage. *J. Plant Physiol.* 171, 31–44. doi: 10.1016/j.jplph.2013.07.014  Hajheidari, M., Abdollahian-Noghabi, M., Askari, H., Heidari, M., Sadeghian, S.Y., Ober, E.S.,and Salekdeh, G.H. (2005). Proteome analysis of sugar beet leaves under drought stress. *Proteomics* 5, 950–960. doi:[10.1002/pmic.200401101](https://doi.org/10.1002/pmic.200401101)  Hradilová, J., Řehulka, P., Řehulková, H., Vrbová, M., Griga, M., and Brzobohatý, B. (2010). Comparative analysis of proteomic changes in contrasting flax cultivars upon cadmium exposure. *Electrophoresis* 31, 421–431. doi: 10.1002/elps.200900477  Komatsu, S. Hiraga, S., and Nouri, M.Z. (2014). Analysis of flooding-responsive proteins localized in the nucleus of soybean root tips. *Mol. Biol. Rep.* 41, 1127–1139. doi: 10.1007/s11033-013-2959-7  Kosmala, A., Bocian, A., Rapacz, M., Jurczyk, B., and Zwierzykowski, Z. (2009). Identification of leaf proteins differentially accumulated during cold acclimation between *Festuca pratensis* plants with distinct levels of frost tolerance. *J. Exp. Bot.* 60, 3595–3609. doi: [10.1093/jxb/erp205](https://doi.org/10.1093/jxb/erp205)  Patterson, J., Ford, K., Cassin, A., Natera, S., and Bacic, A. (2007). Increased abundance of proteins involved in phytosiderophore production in boron-tolerant barley. *Plant Physiol.* 144, 1612–1631. doi: 10.1104/pp.107.096388  Sarhadi, E., Mahfoozi, S., Hosseini, S.A., and Salekdeh, G.H. (2010). Cold acclimation proteome analysis reveals close link between the up-regulation of low-temperature associated proteins and vernalization fulfillment. *J. Proteome Res.* 9, 5658–5667. doi: 10.1021/pr100475r  Sengupta, S., and Majumder, A.L. (2009). Insight into the salt tolerance factors of a wild halophytic rice, *Porteresia coarctata*: a physiological and proteomic approach. *Planta* 229, 911–929. doi: 10.1007/s00425-008-0878-y Vítámvás, P., Saalbach, G., Prášil, I.T., Čapková, V., Opatrná, J., and Ahmed, J. (2007). WCS120 protein family and proteins soluble upon boiling in cold-acclimated winter wheat. *J. Plant. Physiol.*164, 1197–1207. doi: 10.1016/j.jplph.2006.06.011 | | | | |  |

Ye, J., Wang, S., Zhang, F., Xie, D., and Yao, Y. (2013). Proteomic analysis of leaves of different wheat genotypes subjected to PEG 6000 stress and rewatering. *Plant Omics J*. 6, 286–294.

Yin, X.J., and Komatsu, S. (2016). Nuclear Proteomics reveals the role of protein synthesis and chromatin structure in root tip of soybean during the initial stage of flooding stress. *J. Proteome Res.* 15, 2283–2298. doi: 10.1021/acs.jproteome.6b00330

Yu, F., Han, X., Geng, C., Zhao, Y., Zhang, Z., and Qiu, F. (2015). Comparative proteomic analysis revealing the complex network associated with waterlogging stress in maize (*Zea mays* L.) seedling root cells. *Proteomics* 15, 135–147. doi: 10.1002/pmic.201400156
